# Neutrophil Extracellular Traps-Mediated Intraplaque Angiogenesis and Microvascular Permeability Promote Rapid Plaque Progression following Myocardial Infarction

**DOI:** 10.34133/research.1451

**Published:** 2026-10-19

**Authors:** Zhijun Lei, Yunfei Yin, Long Zeng, Na Yang, Ming Zhai, Yanxi Zeng, Yefei Shi, Qing Yu, Shiyu Gong, Weixia Jian, Jianhui Zhuang, Li Zhang, Wenhui Peng

**Affiliations:** ^1^ Department of Cardiology, Shanghai Tenth People’s Hospital , Tongji University School of Medicine, Shanghai, China.; ^2^ Department of Cardiology, Huadong Hospital Affiliated to Fudan University, Shanghai, China.; ^3^ Department of Endocrinology, Xinhua Hospital, Shanghai Jiaotong University School of Medicine, Shanghai, China.; ^4^ Department of Cardiology, Xinhua Hospital, Shanghai Jiaotong University School of Medicine, Shanghai, China.

## Abstract

Despite adherence to guideline-recommended therapies, survivors of myocardial infarction (MI) remain at high risk for recurrent ischemic events. This study aimed to elucidate the underlying mechanisms responsible for this residual risk. Using Mendelian randomization analysis and retrospective cohort study, we established a causal relationship between MI and plaque progression, potentially mediated by neutrophils. In a murine atherosclerotic model, MI precipitated a marked expansion in plaque volume accompanied by reduced stability; these pathological changes are consistent with the hallmark features of rapid plaque progression, which may trigger recurrent ischemic events. Pharmacological inhibition and adoptive transfer experiments using *Padi4^flox/flox^Lyz2^Cre^* mice confirmed the pivotal role of neutrophils in MI-driven rapid plaque progression via neutrophil extracellular traps (NETs) formation. Further investigations revealed that NETs promote intraplaque hemorrhage by facilitating intraplaque angiogenesis and enhancing microvascular permeability, thereby exacerbating post-MI atherosclerotic inflammation. Mechanistically, bioinformatics analyses and in vitro experiments identified the transcription factor ETS proto-oncogene 1 as a critical regulator of NETs-mediated angiogenesis and endothelial permeability through the activation of the vascular endothelial growth factor A/vascular endothelial growth factor receptor 2 signaling pathway. In vivo pharmacological inhibition of ETS proto-oncogene 1 with TK216 markedly attenuated the rapid plaque progression after MI. In summary, this study provides the first comprehensive evidence that neutrophils and NETs drive rapid plaque progression following MI by modulating intraplaque angiogenesis and hemorrhage. These findings offer novel insights into the mechanisms underlying post-MI ischemic event recurrence and highlight promising therapeutic targets for clinical prevention.

## Introduction

Despite advances in cardiovascular medical technology enabling more patients with myocardial infarction (MI) to receive timely and effective treatment, survivors of MI still face an elevated risk of recurrent ischemic events [1,2]. This persistent risk underscores the urgent need to identify the underlying mechanisms. Emerging clinical evidence suggests that rapid plaque progression is a critical precursor to acute ischemic events [3,4]. Pathologically, this rapid progression manifests as swift plaque expansion, frequently accompanied by compromised plaque stability over a short period [3,5,6]. Previous investigations have identified several mechanisms contributing to post-MI plaque progression. In murine models, MI has been shown to promote plaque progression via sympathetic stimulation of bone marrow adrenergic receptors and monocyte mobilization [7], while trained immunity in monocytes [8] and plasma cell–autoantibody–dependent pathways [9] have also been implicated. Collectively, these findings indicate that multiple pathways drive atheroprogression after MI. However, a notable limitation of these studies is their predominant focus on the impact of MI on atherosclerotic lesion burden, with insufficient attention paid to alterations in plaque stability.

Atherosclerosis typically progresses as a chronic inflammatory process but can undergo accelerated progression under specific circumstances [10]. Two distinct progression patterns have been documented: slow linear progression, characterized by gradual plaque growth with maintained stability, typically leading to chronic coronary syndromes; and rapid plaque progression, featuring swift expansion of the necrotic core, which generates advanced, vulnerable atherosclerotic lesions and frequently culminates in plaque rupture and MI [5,11]. Rapid plaque progression likely arise from the interplay between intrinsic plaque characteristics and systemic inflammatory factors. Intrinsically, rapid plaque progression is often driven by subclinical cycles of plaque rupture and healing [12], as well as intraplaque hemorrhage [11,13]. Systemically, acute inflammatory conditions including MI, ischemic stroke, and sepsis have been shown to exacerbate atherosclerosis in both animal models and humans [14–17]. These observations suggest that acute events such as MI can accelerate preexisting atherosclerosis in nonculprit arteries, superimposing upon chronic vascular inflammation.

As central participants in acute inflammation, neutrophils become activated and accumulate in locally injured tissues as well as distant organs during acute inflammatory responses [18,19]. Histological analysis based on 355 human carotid plaques has revealed a positive correlation between neutrophil accumulation and plaque instability, with abundant neutrophils detected in regions of intraplaque hemorrhage and around intraplaque microvessels [20]. However, previous investigations by our group and Grégory Franck et al. have indicated that neutrophils have a negligible effect in conventional atherosclerosis models [21,22]. Nevertheless, whether neutrophils contribute to aggravated atherosclerotic inflammation following MI remains to be determined.

In this study, we establish that atherosclerotic pathological progression in nonculprit lesions following MI recapitulates the hallmark features of rapid plaque progression and demonstrate the key role of neutrophils and neutrophil extracellular traps (NETs) in this process. We further show that NETs promote intraplaque angiogenesis and microvascular permeability, leading to subsequent intraplaque hemorrhage via ETS proto-oncogene 1 (ETS1)-mediated vascular endothelial growth factor A (VEGFA)/vascular endothelial growth factor receptor 2 (VEGFR2) signaling pathway. These findings provide novel mechanistic insights and potential therapeutic avenues to prevent recurrent ischemic events in post-MI patients.

## Results

### Mendelian randomization analyses and retrospective cohort study reveal a causal association between MI and plaque progression in nonculprit arteries, possibly mediated by neutrophils

To investigate the potential causal relationship between MI and atherosclerosis, we performed 2-sample Mendelian randomization (MR) analyses. We identified 42 genetic variants robustly associated with MI (all *F* statistics >10; Table S1). Using the inverse-variance weighted (IVW) method, genetically predicted MI was associated with an increased risk of atherosclerosis (odds ratio [OR] = 1.469, 95% confidence interval [95% CI]: 1.170 to 1.846, *P* < 0.001), coronary atherosclerosis (OR = 1.685, 95% CI: 1.480 to 1.918, *P* < 0.001), atherosclerosis of aorta (OR = 1.960, 95% CI: 1.387 to 2.787, *P* < 0.001), and atherosclerosis of the extremities (OR = 1.718, 95% CI: 1.299 to 2.271, *P* < 0.001) (Fig. 1A). However, no significant association was observed between MI and atherosclerosis of the cerebral or renal arteries (Fig. 1A). Sensitivity analyses using MR Egger, weighted median, simple mode, and weighted mode yielded consistent results (Table S2), supporting a potential causal relationship.

**Fig. 1.**
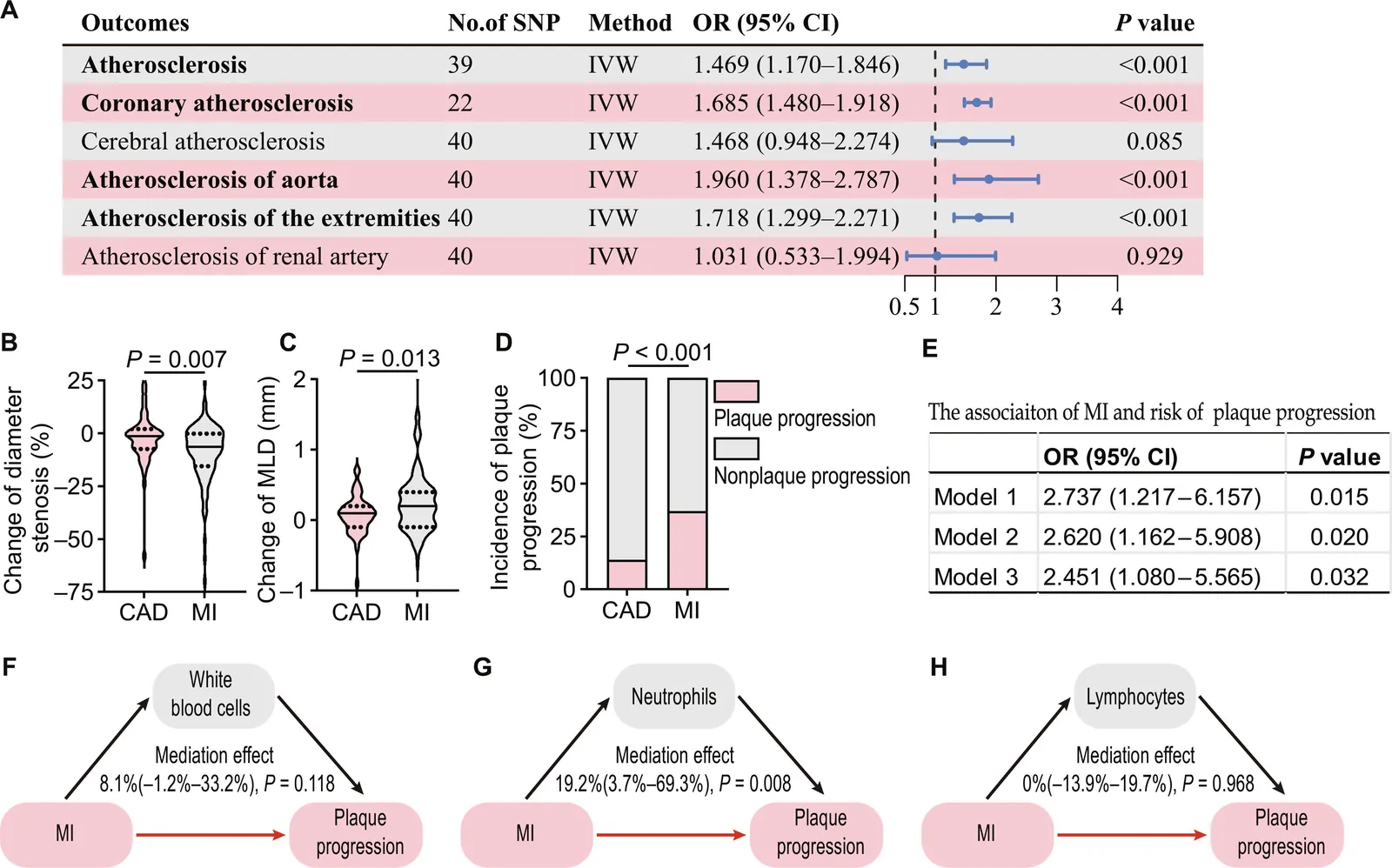
Mendelian randomization (MR) analyses and retrospective cohort study reveal a causal association between myocardial infarction (MI) and plaque progression in nonculprit arteries, possibly mediated by neutrophils. (A) Two-sample MR analysis using the inverse-variance weighted (IVW) method to investigate the association between MI and atherosclerosis. (B to D) Change in diameter stenosis (%), change in minimal lumen diameter (MLD), and incidence of plaque progression in nonculprit arteries in the stable coronary artery disease (CAD) and MI groups from a retrospective cohort study. (E) The association between MI and plaque progression. Model 1, unadjusted; Model 2, adjusted for age and sex; Model 3, adjusted for age, body mass index, smoking, hypertension, history of stroke, and diabetes. (F to H) Mediation analyses to assess the roles of white blood cell count, neutrophil count, and lymphocyte count in the association between MI and plaque progression in nonculprit arteries. Statistical analyses were performed using Mann–Whitney U test (B and C) and chi-square test (D).

To further validate this association, we conducted a retrospective cohort study among patients who underwent percutaneous coronary intervention (PCI) at baseline and had follow-up angiography. We enrolled 150 patients (116 males; median age 66 years), of whom 66.7% were diagnosed with MI and 33.3% had stable coronary artery disease (CAD). Baseline characteristics and angiographic assessments were generally comparable between the 2 groups, except for higher neutrophil count and C-reactive protein level in MI patients (Tables S3 and S4). Compared with the stable CAD group, MI patients exhibited increased diameter stenosis (*P* = 0.007), reduced minimal lumen diameter (MLD) (*P* = 0.015), and a higher incidence of plaque progression (*P* <0.001) in nonculprit arteries during follow-up (Fig. 1B to D and Table S4). After full adjustment for confounders, MI was independently associated with plaque progression in nonculprit arteries during follow-up (adjusted OR: 2.451, 95% CI: 1.080 to 5.565, *P* = 0.032) (Fig. 1E).

Given the involvement of immune cells in both MI pathophysiology and atherosclerotic progression, we performed mediation analyses to assess the potential role of major immune cell subtypes. The results indicated that neutrophil count mediated 19.2% of the association between MI and plaque progression (Fig. 1G). In contrast, no significant mediation effect was observed for white blood cell count or lymphocyte count (Fig. 1F to H). These findings suggest a potential causal link between MI and plaque progression in nonculprit arteries, possibly mediated by neutrophils.

### Increased neutrophil infiltration and NETs formation are associated with rapid plaque progression in nonculprit lesions following MI

To investigate the effects of MI on atherosclerosis in nonculprit arteries, we induced MI in *Ldlr^−/−^* mice that had been fed a high-fat diet (HFD) for 8 weeks, after which they were maintained on the HFD for another 4 or 8 weeks (Fig. 2A). Body weight, plasma lipids, and glucose levels did not differ between MI and sham groups (Fig. S1A and B). En face Oil Red O staining revealed a gradual plaque accumulation in sham-operated mice. By contrast, MI-operated animals exhibited a sharp surge in overall plaque burden at 4 weeks postsurgery (a 30.0% increase in the sham group vs. a 2-fold increase in the MI group). After this early acceleration, plaque expansion slowed markedly in MI mice over the subsequent 4 weeks (a 13.1% increase in the sham group vs. an 11.0% increase in the MI group) (Fig. 2B). A similar change pattern was observed in lesions within the brachiocephalic artery (BCA) (Fig. 2C and D). Masson’s trichrome staining further demonstrated severe impairment of BCA plaque stability in MI mice within the initial 4 weeks postsurgery, corresponding to an ~22% reduction in collagen content, and this collagen deficit remained unchanged over the subsequent 4 weeks, whereas collagen content stayed stable in sham controls (Fig. 2C and E). Collectively, these data showed that MI exerts its dominant phenotypic effects on nonculprit plaques within the first 4 weeks after MI onset. Accordingly, we selected the 4-week postsurgery time point for all subsequent analyses. Beyond BCA lesions, MI also triggered expansion and destabilization of atherosclerotic plaques across the aortic arch, thoracic aorta, and abdominal aorta at 4 weeks post-MI (Fig. S1C). To streamline the analysis, we focused on BCA plaques as representative nonculprit lesions in downstream experiments. Immunofluorescence analysis validated MI-mediated plaque destabilization, showing an ~85% increase in matrix metalloproteinase 9 (MMP9)-positive area and an ~50% decrease in vascular smooth muscle cells (VSMCs) content in MI mice at 4 weeks postsurgery (Fig. S1D to F). Moreover, CD68^+^ macrophage infiltration was 2-fold higher in MI mice than in sham controls at 4 weeks postsurgery, indicating sustained inflammatory activation (Fig. S1D and G). Together, these dynamic pathological changes align with the hallmark features of rapid plaque progression—namely, swift plaque expansion accompanied by compromised stability over a short period [3,5,11]. Therefore, our findings indicate that MI can promote rapid plaque progression in nonculprit lesions, a process that may contribute to the development of recurrent ischemic events.

**Fig. 2.**
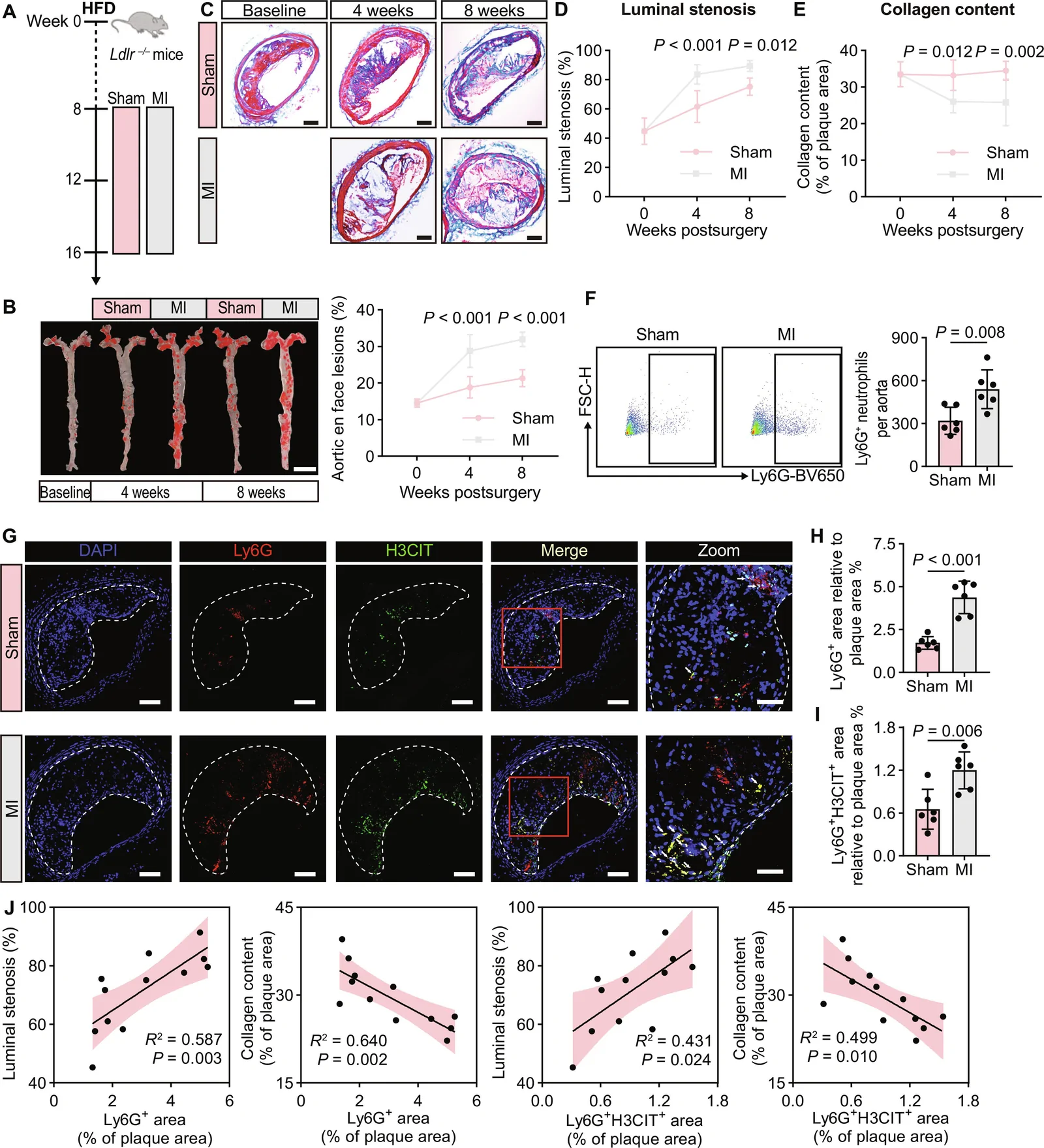
Increased neutrophil infiltration and neutrophil extracellular traps (NETs) formation are associated with rapid plaque progression in nonculprit lesions following myocardial infarction (MI). (A) Schematic of the animal experimental modeling process. (B) Representative images and quantification of en face Oil Red O staining of the aorta from the sham and MI groups (scale bar = 5 mm, *n* = 8 per group). (C) Representative images of Masson’s trichrome staining in brachiocephalic artery (BCA) lesions from the sham and MI groups (scale bar = 100 μm). (D and E) Quantification of luminal stenosis (%) and collagen content in the BCA lesions in (C) (*n* = 6 per group). (F) Flow cytometry analysis and quantification of intraplaque Ly6G^+^ neutrophils in the sham and MI groups at 4 weeks postsurgery (*n* = 6 per group). (G) Representative immunofluorescence images of intraplaque Ly6G and histone H3 citrullination (H3CIT) immunofluorescence staining in the sham and MI groups at 4 weeks postsurgery (scale bar = 100 μm). (H and I) Quantification of Ly6G^+^ area and Ly6G^+^H3CIT^+^ area relative to plaque area in (G) (*n* = 6 per group). (J) Correlation analyses of Ly6G^+^ area and Ly6G^+^H3CIT^+^ area with plaque area and collagen content in BCA lesions. Statistical analyses were performed using 2-way analysis of variance (ANOVA) with Bonferroni posttest (B, D, and E), unpaired Student *t* test (F, H, and I), and Pearson correlation analysis (J).

Consistent with the potential mediating role of neutrophils suggested by retrospective cohort analyses (Fig. 1G), flow cytometry revealed increased intraplaque Ly6G^+^ neutrophils in MI mice (540 ± 135 vs. 319 ± 94 per aorta, *P* = 0.008) (Fig. 2F; gating strategy shown in Fig. S2A). This observation was confirmed by immunofluorescence staining (Fig. 2G and H). NETs formation, assessed by histone H3 citrullination staining, was nearly 2-fold higher in BCA lesions from MI mice than in those from sham controls (Fig. 2G and I). Similarly, increased neutrophil infiltration and NETs formation were observed in atherosclerotic lesions across the aortic arch, thoracic aorta, and abdominal aorta (Fig. S2B). Notably, both intraplaque neutrophil infiltration and NETs formation correlated positively with BCA plaque size and instability (Fig. 2J and Fig. S2C). Taken together, these results indicate that increased neutrophil infiltration and NETs formation are closely associated with the rapid plaque progression in nonculprit lesions following MI.

### Neutrophils mediate rapid plaque progression in nonculprit lesions following MI through NETs formation

To investigate the contribution of neutrophils to rapid plaque progression in nonculprit lesions after MI, we depleted neutrophils in MI-subjected *Ldlr^−/−^* mice using an anti-Ly6G antibody (Fig. 3A). Flow cytometry confirmed efficient neutrophil depletion in peripheral blood (Fig. S3A). Compared with isotype-treated controls, neutrophil depletion markedly attenuated plaque expansion and promoted stability in BCA lesions, as evidenced by increased collagen and VSMCs content, alongside reduced MMP9 levels and CD68^+^ macrophage infiltration (Fig. 3B to D). To specifically assess the role of NETs, we administered deoxyribonuclease I (DNase I) to degrade NETs in vivo (Fig. 3E). DNase I treatment similarly reduced lesion size while improving plaque stability in BCA lesions compared with vehicle-treated mice (Fig. 3F to H).

**Fig. 3.**
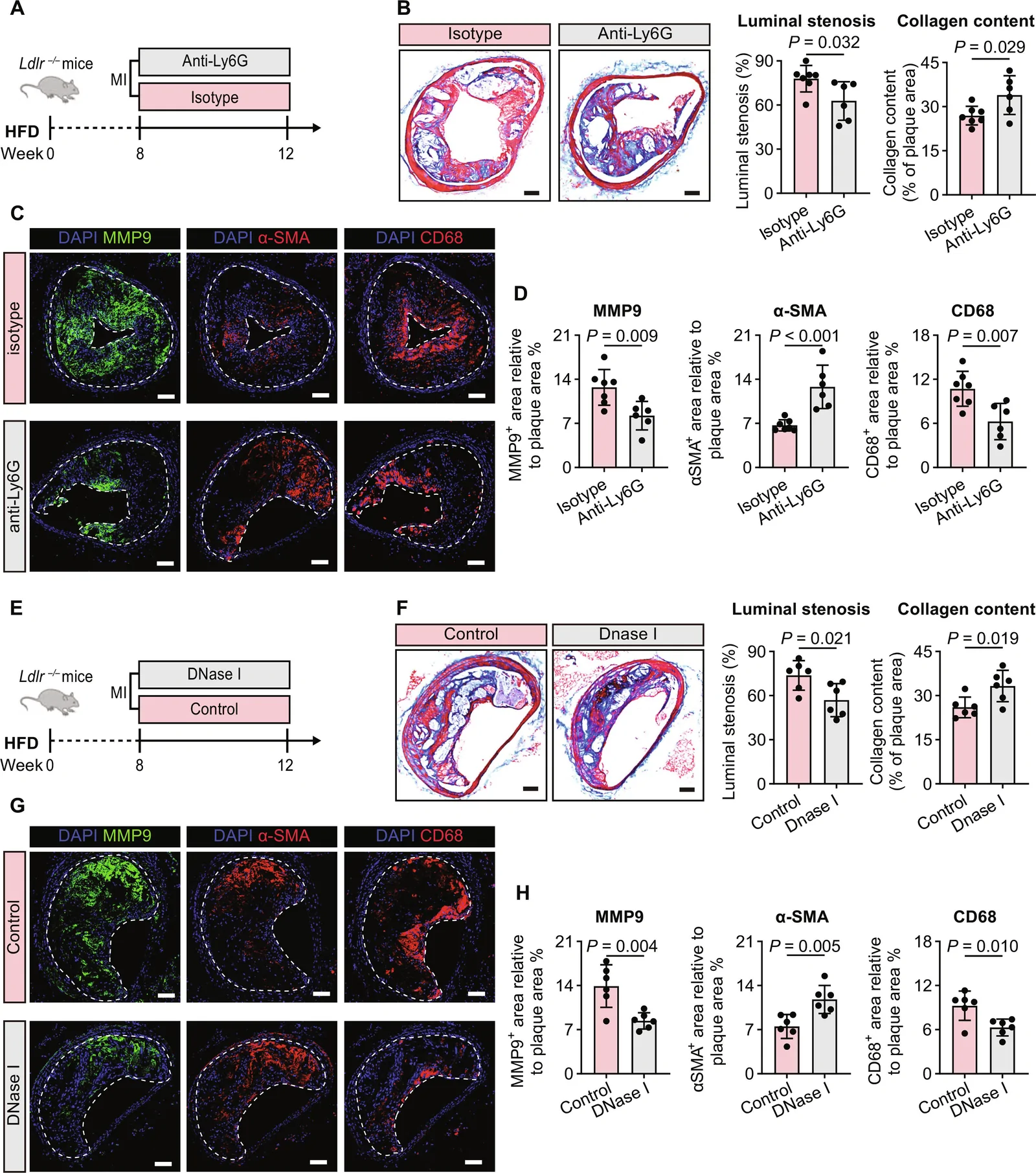
Neutrophils mediate rapid plaque progression in nonculprit lesions following myocardial infarction (MI) through neutrophil extracellular traps (NETs) formation. (A) Schematic of the experimental timeline for neutrophil depletion. (B) Representative Masson’s trichrome staining images and corresponding quantification of luminal stenosis (%) and collagen content in brachiocephalic artery (BCA) lesions from the isotype and anti-Ly6G groups (scale bar = 100 μm, *n* = 6 to 7 per group). (C) Representative images of intraplaque matrix metalloproteinase 9 (MMP9), α-SMA and CD68 immunofluorescence staining in BCA lesions from the isotype and anti-Ly6G groups (scale bar = 100 μm). (D) Quantification of MMP9^+^, α-SMA^+^, and CD68^+^ area relative to plaque area in BCA lesions from the isotype and anti-Ly6G groups in C (*n* = 6 to 7 per group). (E) Flowchart of the animal experimental modeling process for NETs inhibition. (F) Representative Masson’s trichrome staining images and corresponding quantification of luminal stenosis (%) and collagen content in BCA lesions from the control and deoxyribonuclease I (DNase I) groups (scale bar = 100 μm, *n* = 6 per group). (G) Representative images of intraplaque MMP9, α-SMA, and CD68 immunofluorescence staining in BCA lesions from the control and DNase I groups (scale bar = 100 μm). (H) Quantification of MMP9^+^, α-SMA^+^, and CD68^+^ area relative to plaque area in BCA lesions from the control and DNase I groups in G (*n* = 6 per group). Statistical analyses were performed using unpaired Student *t* test (B, D, F, and H).

We further validated these findings through a neutrophil adoptive transfer assay using neutrophils isolated 24 h after sham or MI surgery from *Padi4^flox/flox^* (wild type) and *Padi4^flox/flox^Lyz2^Cre^* (myeloid-specific *Padi4*-deficient, incapable of NETs formation) mice. These neutrophils were delivered onto the adventitia of the BCA lesions of atherosclerotic mice (Fig. S3B). Recipients of *Padi4^flox/flox^* MI neutrophils developed markedly larger lesions with unstable features, including reduced collagen (~25%) and VSMCs content (~50%), elevated MMP9^+^ area (2-fold), and increased CD68^+^ macrophage infiltration (~80%) compared with *Padi4^flox/flox^* sham neutrophil recipients (Fig. S3, C to H). In contrast, genetic ablation of NETs formation in neutrophils abolished these detrimental effects (Fig. S3, C to H). Collectively, these findings demonstrate that neutrophils drive rapid plaque progression in nonculprit lesions following MI primarily through NETs formation.

### NETs-mediated intraplaque hemorrhage drives rapid plaque progression in nonculprit lesions following MI, potentially via macrophage ferroptosis

Pathologically, rapid plaque progression may arise from subclinical cycles of plaque rupture and healing [12] or from intraplaque hemorrhage [11]. To identify the dominant mechanism underlying post-MI rapid plaque progression in nonculprit lesions, we first performed CD31 staining and observed an intact luminal endothelial layer in BCA plaques after MI, indicating the absence of recurrent plaque rupture and healing (Fig. 4A). In contrast, TER119 staining revealed a marked (nearly 4-fold) increase in intraplaque hemorrhage following MI compared with minimal hemorrhage in sham-operated mice (Fig. 4A and B). Increased intraplaque neovascularization density was observed after MI and was spatially adjacent to hemorrhagic areas, suggesting erythrocyte leakage from immature neovessels (Fig. 4A and C). This hemorrhage was accompanied by intraplaque iron deposition, as confirmed by Prussian blue staining (Fig. 4A and D). Notably, intraplaque NETs formation correlated positively with intraplaque hemorrhage and intraplaque iron deposition (Fig. S4A), and NETs inhibition substantially attenuated MI-induced intraplaque hemorrhage (Fig. S4B and C), indicating that NETs contribute to hemorrhage exacerbation post-MI.

**Fig. 4.**
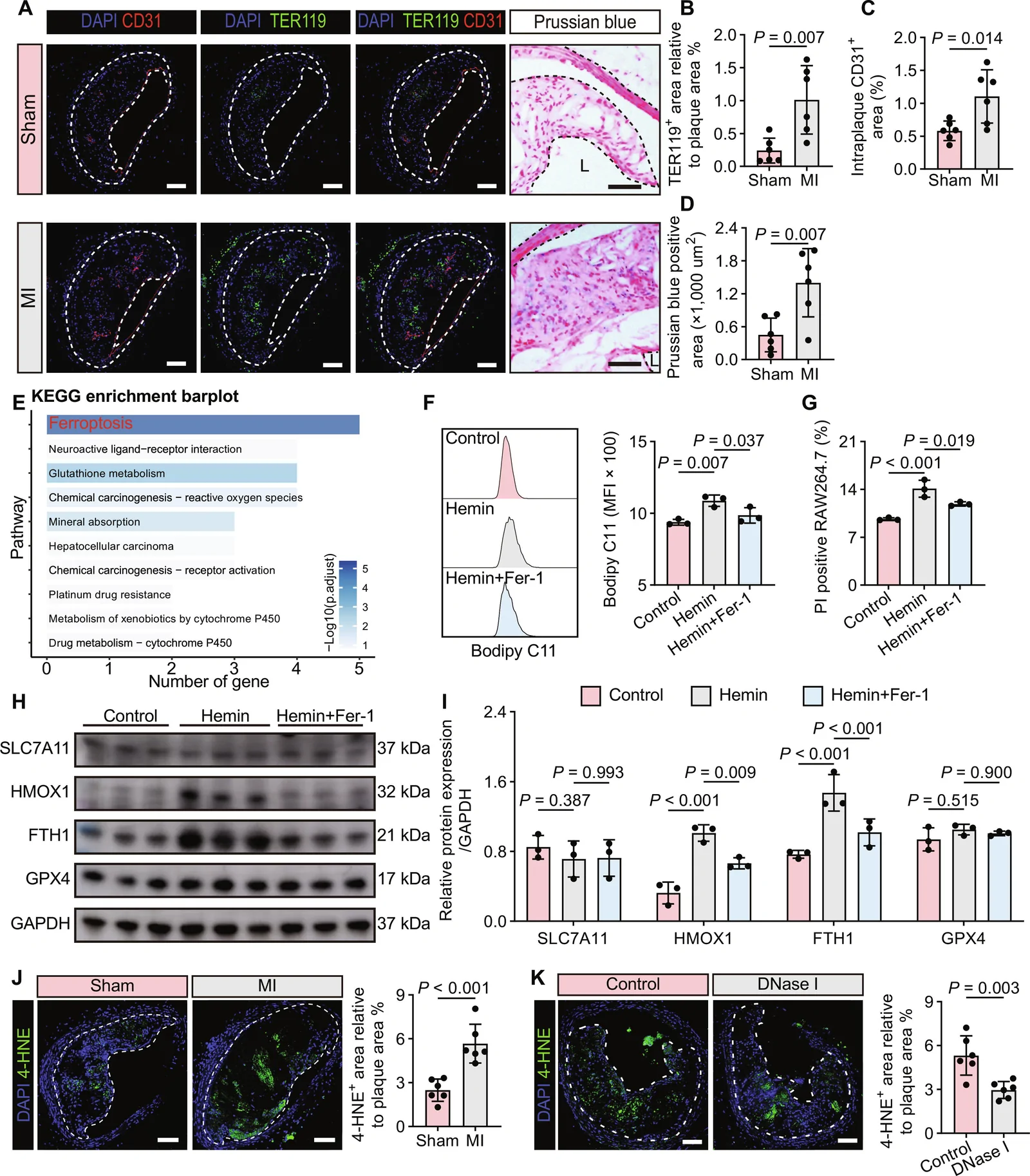
Neutrophil extracellular traps (NETs)-mediated intraplaque hemorrhage drives rapid plaque progression in nonculprit lesions following myocardial infarction (MI), potentially via macrophage ferroptosis. (A) Representative images of intraplaque CD31, TER119 immunofluorescence staining (scale bar = 100 μm), and Prussian blue staining (scale bar = 50 μm) in brachiocephalic artery (BCA) lesions from the sham and MI groups at 4 weeks postsurgery. (B and C) Quantification of TER119^+^ and CD31^+^ area relative to plaque area in (A) (*n* = 6 per group). (D) Quantification of the Prussian blue positive area in (A) (*n* = 6 per group). (E) Kyoto Encyclopedia of Genes and Genomes (KEGG) pathway analysis showing enrichment of the ferroptosis signaling pathway. (F) Quantification of the mean fluorescence intensity (MFI) of Bodipy C11 in RAW264.7 cells across the indicated groups (*n* = 3 per group). (G) Quantification of PI-positive RAW264.7 cells across the indicated groups (*n* = 3 per group). (H and I) Immunoblotting and quantification of the relative protein levels of SLC7A11, HMOX1, FTH1, and GPX4 in RAW264.7 cells across the indicated groups (*n* = 3 per group). (J) Representative images and quantification of 4-hydroxynonenal (4-HNE) immunofluorescence staining in BCA lesions from the sham and MI groups at 4 weeks postsurgery (scale bar = 100 μm, *n* = 6 per group). (K) Representative images and quantification of 4-HNE immunofluorescence staining in BCA lesions from the control and deoxyribonuclease I (DNase I) groups at 4 weeks post-MI (scale bar = 100 μm, *n* = 6 per group). Statistical analyses were performed using unpaired Student *t* test (B, C, D, J, and K) and 1-way analysis of variance (ANOVA) with Bonferroni posttest (F, G, and I).

Intraplaque hemorrhage leads to erythrocyte extravasation into the plaque. Erythrocytes subsequently undergo oxidative damage and are primarily phagocytosed by macrophages, which in turn become proinflammatory [23]. We therefore hypothesized that intraplaque hemorrhage may amplify atherosclerotic inflammation by activating macrophages. To explore the underlying mechanism, we used a published RNA sequencing (RNA-seq) dataset (GSE221734) to analyze differentially expressed genes (DEGs) in bone marrow-derived macrophages stimulated with hemin, a degradation product of erythrocytes commonly used to model hemorrhage (Fig. S4D) [24]. Kyoto Encyclopedia of Genes and Genomes (KEGG) pathway analysis indicated significant enrichment of the ferroptosis signaling pathway (Fig. 4E). In vitro, hemin stimulation promoted ferroptosis in RAW264.7 cells, as indicated by increased lipid peroxidation and cell death, which were abolished by the ferroptosis inhibitor ferrostatin-1 (Fer-1) (Fig. 4F and G). Immunoblotting showed that hemin up-regulated HMOX1 and FTH1 expression without altering GPX4 or SLC7A11 levels (Fig. 4H and I), and Fer-1 suppressed hemin-induced up-regulation of these proteins (Fig. 4H and I). In vivo, atherosclerotic plaques from MI mice exhibited elevated lipid peroxidation and increased macrophage expression of HMOX1 and FTH1 compared with sham controls (Fig. 4J and Fig. S4E and F). Importantly, NETs inhibition markedly reduced these ferroptosis-related markers post-MI (Fig. 4K and Fig. S4G and H). To further verify the role of ferroptosis, we inhibited ferroptosis in MI-treated atherosclerotic mice using Fer-1 (Fig. S5A). The results revealed that ferroptosis inhibition markedly attenuated MI-induced plaque expansion and instability (Fig. S5B to D). Collectively, these findings demonstrate that NETs-mediated intraplaque hemorrhage drives the rapid progression of nonculprit plaques following MI, an effect associated with augmented macrophage ferroptosis.

### NETs promote intraplaque hemorrhage by facilitating intraplaque angiogenesis and microvascular permeability after MI

As shown in Fig. 4A, intraplaque hemorrhage post-MI was associated with elevated intraplaque neovascularization but not with luminal endothelial disruption, suggesting functional heterogeneity among plaque endothelial cells (ECs). Analysis of a human carotid plaque single-cell RNA sequencing (scRNA-seq) dataset (GSE260657) [25] identified 2 EC subpopulations: EC1, expressing ACKR1, KDR, and FABP4 (indicative of a proangiogenic origin); and EC2, characterized by BMX, MGP, and SULF1 (suggestive of a luminal origin) (Fig. S6A to D) [26]. Gene Ontology enrichment analysis showed that genes up-regulated in EC1 were primarily involved in angiogenesis-related pathways, such as EC migration, sprouting angiogenesis, and positive regulation of angiogenesis. In contrast, EC2-enriched genes were associated with arterial development and arterial morphogenesis (Fig. S6E). EC1 also exhibited lower tight junction scores than EC2, indicating higher permeability (Fig. S6F). Analysis of bulk RNA-seq data (GSE163154) further revealed that carotid plaques with intraplaque hemorrhage showed elevated expression of EC1-associated markers (Fig. S6G). These findings suggest that excessive angiogenesis and increased microvascular permeability may contribute to intraplaque hemorrhage.

Given previous reports that NETs can promote plaque destabilization by facilitating intraplaque neovascularization [27], we investigated whether NETs-mediated intraplaque hemorrhage following MI was linked to excessive angiogenesis and microvascular permeability. Since intraplaque neovascularization is not typically observed in the BCA lesions of HFD-fed *Ldlr^−/−^* mice, we employed a tandem stenosis (TS) model that recapitulates unstable plaques with enhanced intraplaque angiogenesis and hemorrhage, as previously described [28]. One week after TS surgery, MI was induced in *Ldlr^−/−^* mice, and atherosclerotic plaques in the TS model were treated as nonculprit lesions for subsequent analyses. Mice were treated with DNase I to inhibit NETs and analyzed after 4 additional weeks of HFD (Fig. 5A). As anticipated, MI resulted in rapid plaque progression in the TS model, manifesting as larger lesions, reduced fibrous cap thickness, decreased VSMCs content, increased MMP9^+^ area, and greater CD68^+^ macrophage infiltration (Fig. S7A to F). MI also increased intraplaque hemorrhage and lipid peroxidation (Fig. S7A, G, and H). All these effects were markedly attenuated by DNase I treatment (Fig. S7A to H). As expected, microvascular density quantified by intraplaque CD31^+^ cells was over 2-fold higher in the MI group than in sham controls, predominantly due to expansion of the EC1 subpopulation (Fig. 5B to D). To assess microvascular permeability, we administered fluorescein isothiocyanate (FITC)-dextran intravenously and observed over a 3-fold greater accumulation of the tracer within plaques of MI mice via confocal microscopy (Fig. 5B and E). Concurrently, expression of vascular endothelial (VE)-cadherin, a key determinant of endothelial junction integrity, was reduced by approximately two-thirds in MI animals (Fig. 5B and F). DNase I treatment normalized these changes, reversing the increases in microvascular density and FITC-dextran leakage and restoring VE-cadherin expression (Fig. 5B to F). These in vivo results indicate that NETs promote intraplaque hemorrhage by increasing intraplaque angiogenesis and microvascular permeability.

**Fig. 5.**
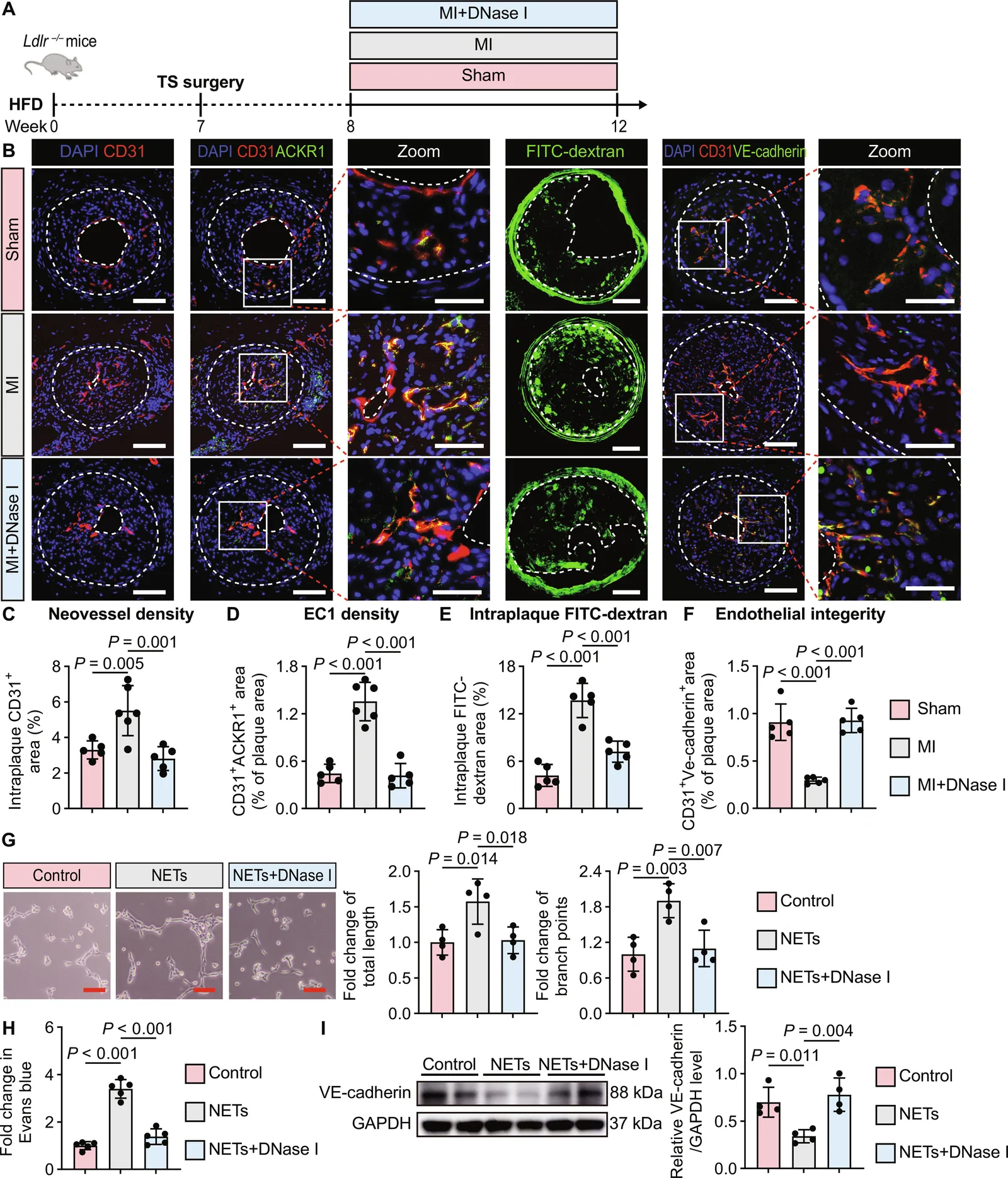
Neutrophil extracellular traps (NETs) promote intraplaque hemorrhage by facilitating intraplaque angiogenesis and microvascular permeability after myocardial infarction (MI). (A) Schematic of the animal experimental modeling process. (B) Representative immunofluorescence staining images of CD31, ACKR1, fluorescein isothiocyanate (FITC)-dextran, and vascular endothelial (VE)-cadherin across the indicated groups (scale bars = 100 μm for regular panels and 50 μm for zoom panels). (C to F) Quantification of intraplaque CD31^+^ area, CD31^+^ACKR1^+^ area, FITC-dextran^+^ area, and CD31^+^VE-cadherin^+^ area relative to plaque area across the indicated groups (*n* = 5~6 per group). (G) Representative images and quantification of the tube formation assay across the indicated groups (scale bar = 50 μm, *n* = 4 per group). (H) Evans-blue cell permeability analysis across the indicated groups (*n* = 5 per group). (I) Immunoblotting and quantification of the relative VE-cadherin protein levels across the indicated groups (*n* = 4 per group). Statistical analyses were performed using 1-way analysis of variance (ANOVA) with Bonferroni posttest (C to I).

To validate these findings in vitro, Human umbilical vein endothelial cells (HUVECs) were exposed to NETs at a gradient of concentrations (0, 0.25, 0.5, 1.0, and 2.0 μg/ml). NETs dose-dependently improved cell viability, proliferation, migration, and tube formation up to the peak dosage of 1.0 μg/ml, followed by weakened facilitatory effects at 2.0 μg/ml (Fig. S8A to D). Regarding endothelial permeability, Evans blue permeability assays revealed that endothelial monolayer leakage increased positively with elevated NETs concentrations (Fig. S8E). In parallel, immunoblotting showed a reduction in VE-cadherin protein abundance upon treatment with NETs at a concentration of 1.0 μg/ml (Fig. S8F). We therefore adopted 1.0 μg/ml NETs for subsequent in vitro assays. Accordingly, DNase I coincubation substantially attenuated NETs-induced augmentation in HUVECs proliferation, migration, and tube formation (Fig. 5G and Fig. S8G and H). Likewise, DNase I reversed NETs-mediated hyperpermeability and VE-cadherin protein loss (Fig. 5H and I). Collectively, our in vitro evidence confirms NETs facilitate angiogenesis and increase vascular permeability. In summary, our results demonstrate that NETs facilitate intraplaque hemorrhage after MI by enhancing intraplaque angiogenesis and microvascular permeability, thereby driving the rapid progression of nonculprit plaques.

### NETs promote angiogenesis and endothelial permeability via ETS1-mediated VEGFA/VEGFR2 signaling pathway

To elucidate how NETs regulate angiogenesis and endothelial permeability, we performed RNA-seq on HUVECs stimulated with NETs (1.0 μg/ml) for 12 h (Fig. S9A and B). Gene Set Enrichment Analysis (GSEA) revealed significant enrichment of the VEGFA/VEGFR2 signaling pathway (Fig. 6A), which is central to angiogenesis and vascular permeability [29]. Immunoblotting confirmed that NETs stimulation increased VEGFA and VEGFR2 protein levels in HUVECs, an effect reversed by DNase I cotreatment (Fig. 6B and C). To further explore how NETs modulate VEGFA/VEGFR2 signaling, we identified ETS1 as the most strongly up-regulated transcript upon NETs stimulation by intersecting DEGs from NETs-stimulated HUVECs with those defining the proangiogenic EC1 subcluster (Fig. 6D and E). ETS1 is a key transcriptional regulator of angiogenesis [30], yet its role in atheroprogression and plaque stability remains unclear. Its expression was markedly increased in plaques of MI mice and in NETs-treated HUVECs, and these increases were attenuated by NETs inhibition (Fig. 6B and C and Fig. S9C). Functionally, small interfering RNA (siRNA)-mediated knockdown of ETS1 in HUVECs abolished the NETs-induced up-regulation of VEGFA and VEGFR2 and impaired endothelial proliferation, migration, and tube formation, while also reducing endothelial permeability (Fig. 6F to J and Fig. S9D and E). In summary, these findings demonstrate that NETs promote angiogenesis and increase endothelial permeability through ETS1-mediated activation of the VEGFA/VEGFR2 signaling pathway.

**Fig. 6.**
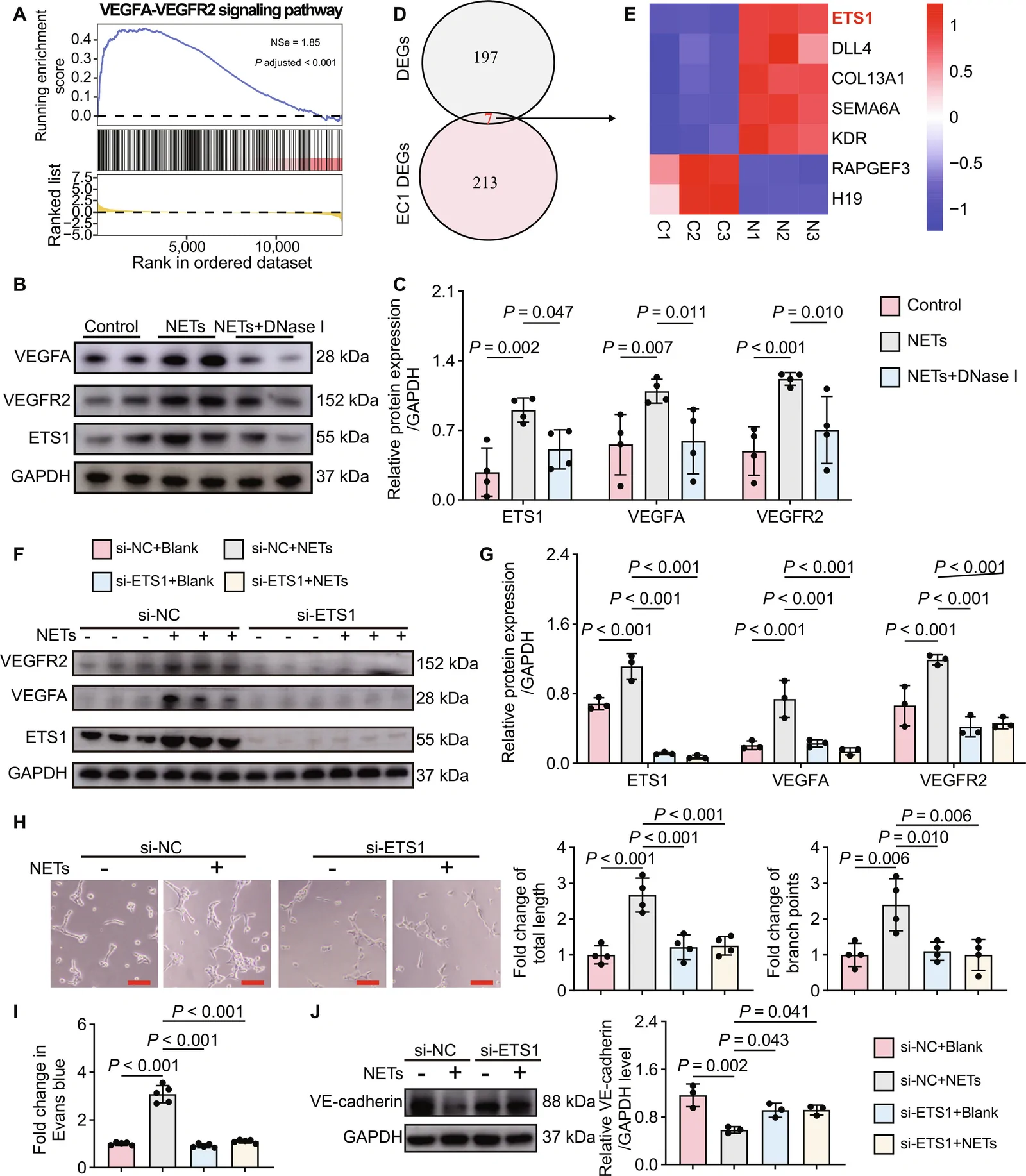
Neutrophil extracellular traps (NETs) promote angiogenesis and endothelial permeability via ETS proto-oncogene 1 (ETS1)-mediated vascular endothelial growth factor A (VEGFA)/vascular endothelial growth factor receptor 2 (VEGFR2) signaling pathway. (A) Gene Set Enrichment Analysis (GSEA) of the differentially expressed genes (DEGs) from NETs-stimulated human umbilical vein endothelial cells (HUVECs). (B and C) Immunoblotting and quantification of the relative protein levels of VEGFA, VEGFR2, and ETS1 across the indicated groups (*n* = 4 per group). (D and E) Overlap between DEGs from the EC1 subcluster and those from NETs-stimulated HUVECs. (F and G) Immunoblotting and quantification of the relative protein levels of VEGFA, VEGFR2, and ETS1 across the indicated groups (*n* = 3 per group). (H) Representative images and quantification of the tube formation assay across the indicated groups (scale bar = 50 μm, *n* = 4 per group). (I) Evans-blue cell permeability analysis across the indicated groups (*n* = 5 per group). (J) Immunoblotting and quantification of the relative protein levels of vascular endothelial (VE)-cadherin across the indicated groups (*n* = 3 per group). Statistical analyses were performed using 1-way analysis of variance (ANOVA) with Bonferroni posttest (C, G, H, I, and J).

### Pharmacological inhibition of ETS1 attenuates rapid plaque progression in nonculprit lesions following MI

To evaluate the translational potential of targeting ETS1, we employed its small-molecule inhibitor TK216, which represses downstream gene expression by disrupting protein–protein interactions between ETS transcription factors and transcriptional coregulators or additional proteins [31,32]. In vitro, TK216 effectively suppressed the NETs-induced up-regulation of ETS1, VEGFA, and VEGFR2 in HUVECs (Fig. S10A). Chromatin immunoprecipitation (ChIP)-quantitative polymerase chain reaction (qPCR) assays further demonstrated that TK216 treatment substantially attenuated the enrichment of ETS1 at the promoter regions of both VEGFA and VEGFR2 following NETs stimulation (Fig. S10B). Moreover, TK216 treatment inhibited proliferation, migration, and tube formation in HUVECs, while enhancing barrier function (Fig. S10C to G). These findings indicate that TK216 counteracts NETs-driven angiogenesis and endothelial permeability by blocking the ETS1-mediated VEGFA/VEGFR2 pathway, supporting its potential as a therapeutic candidate.

We next validated these findings in vivo using the TS model in *Ldlr^−/−^* mice. MI was induced 1 week after TS surgery, followed by TK216 administration (Fig. 7A). TK216 markedly attenuated MI-induced lesion expansion and plaque destabilization, reflected by reduced luminal stenosis and increased collagen content (Fig. S11A to C). Furthermore, TK216 elevated VSMCs content while reducing MMP9^+^ area and CD68^+^ macrophage infiltration (Fig. S11A and D to F). Intraplaque hemorrhage and lipid peroxidation were also markedly reduced by TK216 treatment (Fig. S11A and G and H). Notably, TK216 decreased intraplaque neovessel density and the proportion of EC1 cells, indicating suppression of intraplaque angiogenesis (Fig. 7B to D). In addition, TK216 reduced FITC-dextran extravasation and increased VE-cadherin expression, suggesting improved microvascular integrity (Fig. 7B, E, and F). Together, these data show that pharmacological inhibition of ETS1 with TK216 attenuates post-MI rapid plaque progression in nonculprit lesions by mitigating NETs-induced intraplaque angiogenesis and vascular permeability.

**Fig. 7.**
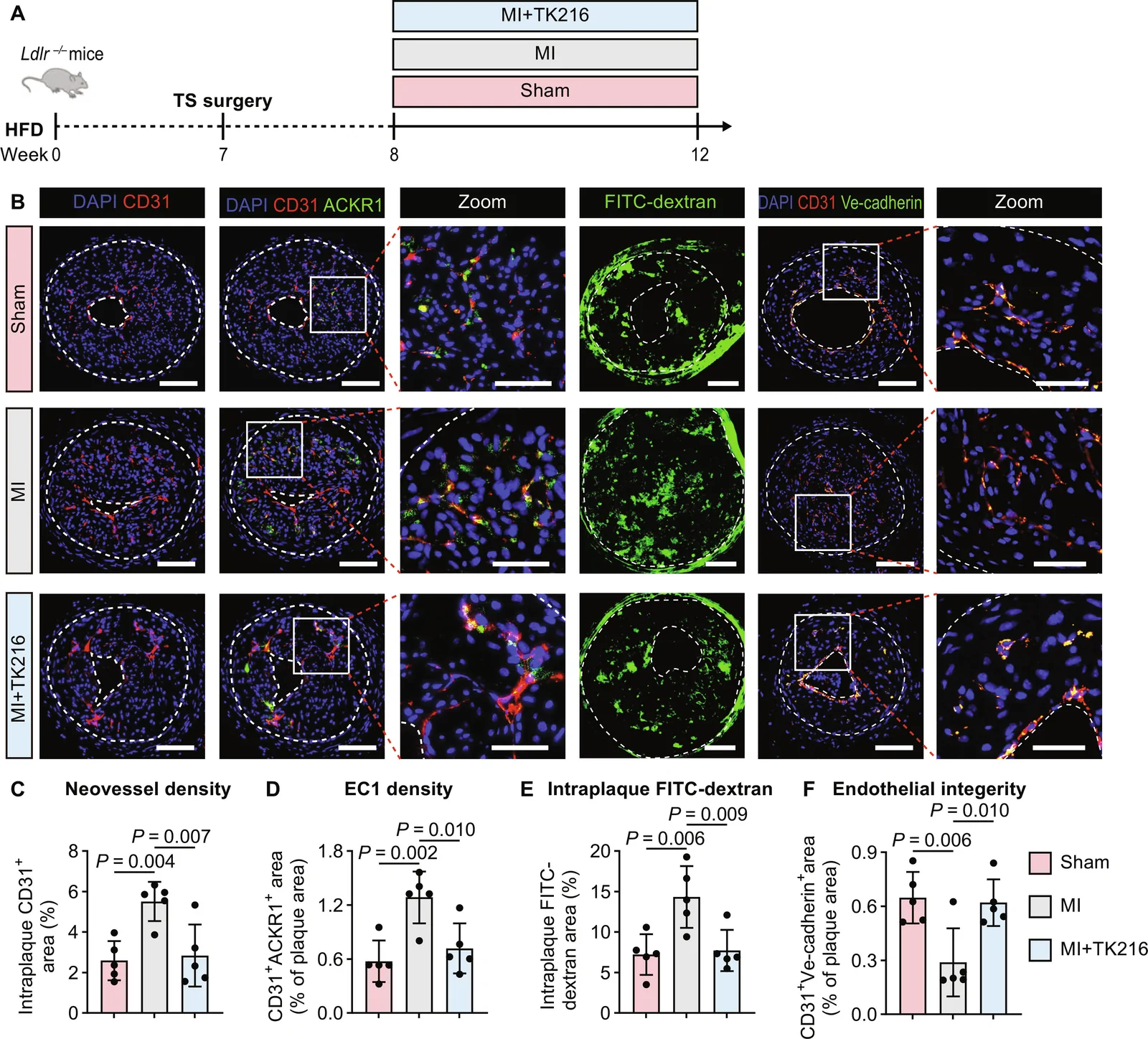
Pharmacological inhibition of ETS proto-oncogene 1 (ETS1) attenuates rapid plaque progression in nonculprit lesions following myocardial infarction (MI). (A) Schematic of the animal experimental modeling process. (B) Representative immunofluorescence staining images of CD31, ACKR1, fluorescein isothiocyanate (FITC)-dextran and vascular endothelial (VE)-cadherin across the indicated groups (scale bars = 100 μm for regular panels and 50 μm for zoom panels). (C to F) Quantification of intraplaque CD31^+^ area, CD31^+^ACKR1^+^ area, FITC-dextran^+^ area, and CD31^+^Ve-cadherin^+^ area relative to plaque area across the indicated groups (*n* = 5 per group). Statistical analyses were performed using 1-way analysis of variance (ANOVA) with Bonferroni posttest (C to F).

## Discussion

Our study reveals that MI promotes rapid plaque progression in nonculprit arteries, a process that facilitates recurrent ischemic events. Mechanistically, we identify the transcription factor ETS1 as a key regulator that activates the VEGFA/VEGFR2 signaling pathway in ECs in response to elevated NETs formation post-MI. This ETS1-mediated signaling promotes the formation of immature, hyperpermeable neovessels, culminating in intraplaque hemorrhage and subsequent rapid plaque progression (Fig. 8). These findings provide novel therapeutic targets for preventing recurrent ischemic events in post-MI patients.

**Fig. 8.**
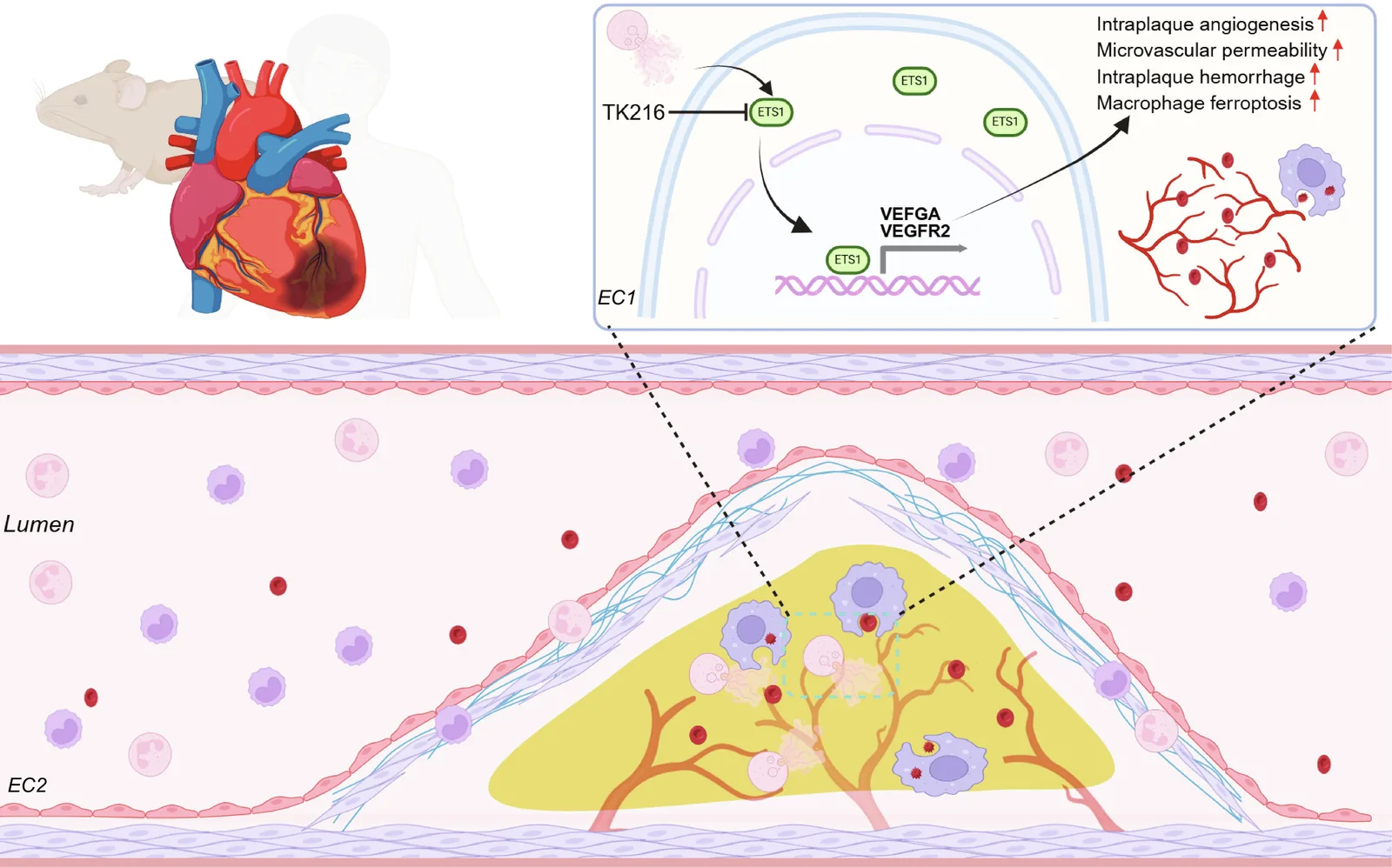
Mechanism diagram. Myocardial infarction (MI) promotes nonculprit plaque rapid progression, a process that facilitates recurrent ischemic events. Mechanistically, we identify the transcription factor ETS proto-oncogene 1 (ETS1) as a key regulator that activates the vascular endothelial growth factor A (VEGFA)/vascular endothelial growth factor receptor 2 (VEGFR2) signaling pathway in ECs in response to elevated neutrophil extracellular traps (NETs) formation post-MI. This ETS1-mediated signaling promotes the formation of immature, hyperpermeable neovessels, culminating in intraplaque hemorrhage and subsequent rapid plaque progression in nonculprit plaques.

The VALIANT study demonstrated that the risk of recurrent ischemic events rises sharply within the first month after MI and gradually declines over the following 3 months [1]. While such events have traditionally been ascribed to the linear progression of nonculprit plaques, emerging clinical evidence suggests that rapid plaque progression can occur as early as 1 to 3 months before an MI, potentially serving as a critical link between early atherosclerosis and subsequent cardiovascular events [33]. Notably, this window of rapid plaque progression coincides with the period of heightened risk for recurrent ischemic events post-MI, supporting the hypothesis that rapid plaque progression in nonculprit arteries contributes to these recurrences. Although previous experimental studies examined the impact of MI on inflammatory responses in preexisting atherosclerosis, most focused primarily on changes in atherosclerotic lesion burden [7–9]. In contrast, our study highlighted that MI not only rapidly expanded plaque volume but also markedly compromised plaque stability—as evidenced by reduced collagen and VSMCs content, elevated MMP9 levels, and increased macrophage infiltration. Importantly, these dynamic pathological changes align closely with the defining features of rapid plaque progression observed clinically: swift expansion coupled with structural destabilization over a short period [3,5]. Thus, our findings support the concept that MI promotes rapid plaque progression in nonculprit lesions, offering a plausible pathological explanation for the elevated risk of recurrent ischemic events in MI survivors.

Both preclinical and clinical evidence indicate that atherosclerosis progression is modulated by interactions between systemic factors and the local microenvironment [34,35]. While atherosclerosis typically progresses as a chronic inflammatory process, it can undergo accelerated progression under specific conditions, such as acute inflammation [10]. In this study, we demonstrated that MI markedly promoted rapid plaque progression in nonculprit lesions, suggesting that superimposition of acute inflammation upon chronic vascular inflammation drives disease advancement. Notably, this accelerated progression was associated with substantial neutrophil infiltration and NETs formation within plaques. Previous studies from our team and the work reported by Grégory Franck et al. [21,22] have shown minimal involvement of neutrophils and NETs in conventional atherosclerosis models, as neutrophil infiltration diminishes with plaque progression. In contrast, our data reveal significant reaccumulation of activated neutrophils and NETs formation within preexisting plaques in the MI-elicited acute inflammatory environment, which indicates inflammatory stimuli promote neutrophil recruitment into established atherosclerotic lesions. This observation aligns with prior reports demonstrating that endotoxemia induces neutrophil infiltration and activation within atherosclerotic lesions [36,37]. Given neutrophils’ short lifespan, these cells cannot directly sustain the long-term proatherogenic effects triggered by MI. Further mechanistic investigations reveal that NETs drive rapid plaque progression post-MI by exacerbating intraplaque angiogenesis and hemorrhage, and subsequent macrophage ferroptosis. We show that under acute inflammatory conditions following MI, NETs interact with resident vascular cells to initiate a pathological signaling cascade. This cascade amplifies vascular inflammation, accelerates lesion expansion, and facilitates plaque destabilization. We therefore propose that inhibiting upstream steps of this cascade—such as NETs formation or intraplaque angiogenesis—could effectively mitigate atherosclerotic inflammatory responses.

Regarding the mechanism by which NETs mediate rapid plaque progression after MI, our study highlights their crucial role in promoting intraplaque angiogenesis and hemorrhage. Although subclinical cycles of plaque rupture and healing are very common and represent another pathway for rapid disease progression [12], our analysis uniquely confirmed the absence of recurrent plaque rupture in nonculprit plaques following MI, as indicated by preserved luminal ECs integrity (Fig. 4A). Moreover, whereas our previous work highlighted the role of VSMCs-derived fibroblast-like cells in plaque stabilization and healing rather than neutrophils or NETs [38], the current study uncovered a distinct inflammatory mechanism that operates under acute post-MI conditions. Pathologically, intraplaque angiogenesis is a hallmark of advanced atherosclerosis [39] and is strongly associated with adverse cardiovascular outcomes [40]. Through scRNA-seq analysis of human carotid plaques, we identified 2 EC subpopulations: one of luminal origin, marked by arterial developmental genes, and a proangiogenic cluster expressing ACKR1, which spatial transcriptomics has recently localized to adventitial sources [41]. This supports the prevailing view that adventitial vasa vasorum represent the primary source of intraplaque neovessels [42]. Although NETs have established proangiogenic roles in pulmonary hypertension, cancer, and corneal neovascularization [43–45], their function in atherosclerosis remains incompletely understood. Building on work by Cao et al. [27], who reported NETs-induced neovascularization in *APOE^−/−^* mice, our study extends this paradigm by demonstrating that NETs drive expansion of a specific proangiogenic EC1 subcluster following MI, using both bioinformatic analyses and a TS model of unstable plaques. Furthermore, these NETs-induced neovessels are structurally immature, deficient in VE-cadherin, and hyperpermeable, thereby promoting intraplaque hemorrhage. As an established independent risk factor for plaque progression and rupture [40,46–48], intraplaque hemorrhage introduces erythrocyte-derived cholesterol, phospholipids, and iron-rich products into lesions, thereby accelerating atheroma growth and undermining plaque stability [49]. Previous studies have demonstrated that intraplaque hemorrhage promotes plaque progression and destabilization via macrophage ferroptosis [23,50]. Consistently, our findings reveal that intraplaque hemorrhage-driven rapid progression of nonculprit plaques after MI is associated with activation of the noncanonical macrophage ferroptosis pathway, corroborating prior reports [23]. In summary, these results establish NETs-driven intraplaque angiogenesis and subsequent hemorrhage as critical mechanisms in post-MI nonculprit plaque rapid progression.

Preclinical studies have established that suppression of intraplaque angiogenesis can alleviate atherosclerosis and promote plaque stabilization [51,52]. However, clinically available angiogenesis inhibitors—primarily used in oncology—are associated with cardiovascular toxicities, including hypertension, thrombosis, and left ventricular dysfunction [53], highlighting the need to identify more specific regulators of atherosclerosis-related angiogenesis. In the present study, we identified the transcription factor ETS1 as a key driver of intraplaque angiogenesis and microvascular permeability following MI. As a member of the ETS transcription factor family, ETS1 is known to regulate vascular development. Recent evidence indicates that ETS1 deficiency down-regulates proangiogenic genes [54], and several studies highlight its involvement in endothelial-to-mesenchymal transition [55–57]. Additionally, ETS1 down-regulation has been observed in cardiac vascular ECs under diabetic conditions [58]. Despite these insights, its role in atherosclerosis progression and plaque stability has remained unclear. Our research unveils a novel mechanism in which ETS1, particularly within an intraplaque EC subcluster (EC1) rather than the luminal EC subcluster (EC2), mediates NETs-activated VEGFA/VEGFR2 signaling after MI. This signaling cascade promotes intraplaque angiogenesis and enhances microvascular permeability, leading to hemorrhage that exacerbates local inflammation and accelerates plaque progression. Translationally, we identified the small-molecule ETS1 inhibitor TK216 as a promising therapeutic candidate. Using a combination of in vitro and in vivo models, we demonstrated that TK216 effectively attenuates rapid plaque progression after MI. These results suggest that targeted inhibition of ETS1 may represent a novel and well-tolerated strategy for reducing the risk of recurrent ischemic events in post-MI patients.

We also acknowledge several limitations in this study. Firstly, our study indicates that intraplaque NETs formation drives rapid progression of nonculprit lesions following MI, yet this conclusion is predominantly supported by preclinical rather than direct human evidence. Prior reports have noted that NETs are rarely detected in atherosclerotic plaques and are largely found on the surface of eroded plaques or in the thrombi overlying ruptured plaques [22,59]. Consistent with these observations, our study only implies increased NETs formation in nonculprit plaques under the acute-inflammatory milieu triggered by MI. While direct human evidence for intraplaque NETs remains lacking, 2 lines of indirect, human-derived data lend support to our hypothesis. A histological study of 355 human carotid plaque specimens documented abundant neutrophil accumulation within intraplaque hemorrhage regions and adjacent to intraplaque microvessels. In addition, single-sample GSEA performed on the RNA-seq dataset (GSE163154) demonstrates elevated NETs-related score in hemorrhagic plaques; this score is positively correlated with EC1 endothelial and plaque-destabilization markers but shows no significant correlation with EC2 markers (Fig. S12A and B). Collectively, these human-based findings constitute indirect evidence for our central hypothesis that NETs drive post-MI nonculprit lesion progression by modulating intraplaque angiogenesis and hemorrhage. Secondly, we observed marked translational gaps in intraplaque angiogenesis between mouse models and humans, including disparities in inflammatory profiles, hemodynamics, shear stress, and vessel integrity. Nevertheless, a key limitation of the mouse TS model employed here is that, while it recapitulates features of plaque instability, it does not lead to clinical cardiovascular events such as MI or stroke. Thus, the model does not fully capture the consequences of lesion instability beyond histopathological indicators [60,61]. Future studies validating these findings in other experimental settings or larger animal models would be of great value. Finally, although the ETS1 inhibitor TK216 demonstrated promising results in alleviating MI-accelerated atherosclerosis, its translational relevance must be confirmed in human studies. Despite this promise, the compelling therapeutic rationale for targeting ETS1—supported by our findings that it mitigates NETs-induced angiogenesis and permeability—mandates rigorous preclinical validation and clinical trials of TK216 and analogous agents prior to any therapeutic use.

In conclusion, this research provides valuable insights into the cellular and molecular mechanisms of MI-induced rapid plaque progression in nonculprit lesions, opening new possibilities for targeted therapies.

## Materials and Methods

### Clinical study

A retrospective study was conducted at the Department of Cardiology of Shanghai Tenth People’s Hospital from January 2020 to June 2021. Patients who underwent PCI at baseline and had follow-up coronary angiography (≥3 months) were consecutively screened. Exclusion criteria were as follows: (a) age < 18 years; (b) presence of severe infections; (c) estimated glomerular filtration rate ≤ 30 ml/min/1.73 m^2^; (d) severe hepatic insufficiency, defined as alanine aminotransferase > 3 times the upper limit of normal; (e) diagnosis of malignant tumors. Enrolled patients were categorized into 2 groups: those with MI (*n* = 100) and those with stable CAD (*n* = 50). Demographic characteristics were collected. Blood samples were drawn from the cubital vein after at least 8 h of fasting following admission and analyzed in a central laboratory. MI was diagnosed in accordance with contemporary guidelines [62]. Quantitative coronary analysis (Centricity Cardiology CA 1000 v1.0, USA) was performed using coronary angiography images recorded at identical projections during baseline and follow-up assessments. To evaluate the impact of MI on nonculprit arteries, all coronary arteries treated with PCI were excluded from analysis. Only coronary artery segments with a reference diameter ≥ 1.5 mm and new lesions identified at follow-up were analyzed. Changes in quantitative coronary analysis measurements were calculated by subtracting follow-up values from baseline values. The lesion with the most significant reduction in MLD was analyzed and recorded for each patient. Plaque progression was defined as the presence of ≥ 1 lesion with a decrease in MLD ≥ 0.4 mm from baseline to follow-up angiography, as previously described [6]. This study conformed to the principles of the Declaration of Helsinki and was approved by the Ethical Committee of Shanghai Tenth People’s Hospital (Approval No. 24KN73). Informed consent was waived due to the retrospective nature of the study.

### MR analyses

Two-sample MR analyses were conducted to explore the causal effect of MI on atherosclerosis. Genome-wide association study (GWAS) summary data for MI were obtained from the FinnGen consortium (https://r10.finngen.fi/), comprising 26,060 cases and 343,079 controls. Instrumental variables for atherosclerosis phenotypes were obtained from GWAS Catalog [63], including atherosclerosis (GCST90436147), coronary atherosclerosis (GCST90436075), cerebral atherosclerosis (GCST90436140), atherosclerosis of aorta (GCST90436150), atherosclerosis of the extremities (GCST90436149), and atherosclerosis of renal artery (GCST90436148).

Instrumental variable selection was based on 3 key assumptions: relevance, independence, and exclusion restriction. To identify single-nucleotide polymorphisms (SNPs) significantly associated with MI (the exposure), a strict threshold was applied (*P* < 5e−8, kb = 10,000, *r*^2^ < 0.001). A total of 44 SNPs were selected as instrumental variables for MI. The strength of the association between each SNP and the exposure was assessed using the *F* statistic; all selected SNPs (*F* > 10) demonstrated a strong association with MI. During harmonization, 2 palindromic SNPs (rs12190287 and rs1556516) were excluded. The remaining SNPs were used in the subsequent MR analyses (Table S1).

### Animals

Male *Ldlr^−/−^*, *Padi4^flox/flox^* (purchased from Cyagen Biosciences), *Lyz2^Cre^* (purchased from Nan Jing Xietong Biotechnology company) mice were used in this study. *Padi4^flox/flox^* mice were crossbred with *Lyz2^Cre^* mice to generate *Padi4^flox/flox^Lyz2^Cre^* mice. *Padi4^flox/flox^* littermates served as controls. All mice were housed under a 12-h light/dark cycle in a temperature-controlled environment (18 to 25 °C). At the start of the experiments, mice were 6 to 8 weeks old. Atherosclerosis was induced in *Ldlr^−/−^* mice by feeding them a Clinton/Cybulsky High Fat Rodent Diet (HFD; D12108C, Research Diets), which provides 40% of calories from fat and contains 1.25% cholesterol. All animal procedures were approved by the Animal Care and Use Committee of Shanghai Tenth People’s Hospital (Approval No. SHDSYY-2024-X0076) and were conducted in accordance with the Guide for the Care and Use of Laboratory Animals published by the National Institutes of Health (NIH Publication, 8^th^ Edition, 2011).

### MI surgery

MI was induced as previously described [8]. Briefly, mice were anesthetized with amobarbital (60 mg/kg, intraperitoneal injection) and mechanically ventilated. A 1.2-cm skin incision was made over the left chest, and the left anterior descending (LAD) coronary artery was ligated at its emergence from the left atrium using a 6-0 silk suture. Complete LAD occlusion was confirmed by visual observation of myocardial blanching in the perfusion bed. Sham-operated animals underwent identical surgical procedures except that the suture was passed under, but not tied around, the LAD. Mice that recovered fully from surgery were returned to standard housing and maintained on a HFD for 4 weeks or 8 weeks a as indicated in the experimental design. Animals were randomly assigned to treatment groups. Mice that died or became severely moribund during or shortly after surgery were excluded from the study.

### TS model

To establish an animal model suitable for studying intraplaque angiogenesis, we employed a TS model of unstable plaque, following our previous research [38]. Male *Ldlr^−/−^* mice were fed a HFD for a total of 12 weeks. The experimental timeline was as follows: after 7 weeks of HFD feeding, TS surgery was performed; 1 week after TS surgery, mice underwent either MI or sham operation; tissues were collected 5 weeks after TS surgery. For the TS procedure, mice were anesthetized with amobarbital (60 mg/kg, intraperitoneal injection), and a sagittal anterior neck incision was made to expose the right common carotid artery. Two stenoses with an outer diameter of 150 μm were created: a distal stenosis 1 mm from the carotid bifurcation and a proximal stenosis 3 mm upstream from the distal site. Stenosis was induced by tying a 6-0 polyester suture around the artery alongside a 150-μm needle, which was subsequently removed. The 5-week post-TS time point for tissue collection was chosen based on prior validation studies indicating that plaque rupture and thrombosis are rare at this stage [60], thereby minimizing their confounding effects on the assessment of intraplaque hemorrhage and angiogenesis.

### Pharmacological in vivo treatment

For neutrophil depletion, animals received intraperitoneal injections of a mouse anti-Ly6G antibody (clone 1A8, BioXcell) at a dose of 200 μg every 48 h for 4 weeks [64]. The first dose was administered 1 d before MI induction. To inhibit NETs formation in vivo, mice received intravenous injections of 400-U ribonuclease-free DNase I (from bovine pancreas, Sigma D7291) in 200 μl of phosphate-buffered saline (PBS), or PBS alone as a control, 3 times weekly for 4 weeks [65]. The first DNase I dose was administered 8 h before MI [65]. To inhibit ferroptosis, mice were treated with intraperitoneal injections of Fer-1 (5 mg/kg/d; MCE, HY-100579) for 4 weeks following MI, with the initial dose administered 8 h prior to MI induction [66]. To inhibit ETS1, mice were treated with the small-molecule inhibitor TK216 (MCE, HY-122903) via oral gavage (100 mg/kg, twice daily) during the final 4 weeks of the protocol [67]. The first TK216 dose was administered 8 h before MI induction.

### Neutrophil adoptive transfer assay

Neutrophils were isolated from the peripheral blood of *Padi4^flox/flox^* and *Padi4^flox/flox^Lyz2^Cre^* mice 1 d after sham or MI surgery using the MojoSort Mouse Neutrophil Isolation Kit (480058, BioLegend) according to the manufacturer’s instructions. Briefly, non-neutrophils were labeled with a biotin-antibody cocktail and subsequently bound to streptavidin-coated magnetic beads. The unlabeled neutrophil fraction was then collected for further use. The purity of the isolated neutrophils exceeded 98%, and viability, assessed by trypan blue exclusion, was greater than 97%. Neutrophil adoptive transfer assay was performed according to a previous study with minor modifications [36]. Briefly, atherosclerotic *Ldlr^−/−^* mice were anaesthetized, and the BCA was carefully dissected from surrounding tissues. To provoke neutrophil plaque invasion, 10 μl of isolated neutrophils at 2 × 10^8^ cells/μl in PBS were pipetted onto the adventitia of the exposed arteries. Four weeks after neutrophil delivery, the BCA was carefully harvested for subsequent analyses.

### Analyses of atherosclerotic lesions

At the end of the indicated treatment periods, mice were anesthetized and sacrificed. Blood was collected into heparin-coated tubes by cardiac puncture. Animals were then perfused with 5 ml of PBS, followed by 10 ml of 4% paraformaldehyde, and finally with 5 ml of PBS. The entire aorta was cleared of surrounding fat and connective tissue, and fixed in 4% paraformaldehyde. To mimic the impact of MI on nonculprit arteries in clinical practice, lesions in the BCA and the segment proximal to the proximal stenosis of the right carotid artery of the TS model were treated as nonculprit lesions. These specimens were carefully dissected, embedded in optimal cutting temperature compound, frozen, sectioned, and subjected to further analysis. For en face Oil Red O staining of whole aorta, fixed aortas were washed in PBS, incubated in Oil Red O solution for 1 h at room temperature, and then washed 3 times with 60% isopropanol followed by PBS on a shaker. The aortas were pinned onto a silicon elastomer pad and imaged using widefield microscopy. Plaque lesion size and collagen content were quantified on sections using Masson’s trichrome staining. Intraplaque hemorrhage was detected using Prussian blue staining. All image analyses were performed using ImageJ software (V2.10, NIH, USA).

### Plasma index measurement

Plasma was collected from the mice at the time of sacrifice. Concentrations of total triglycerides, total cholesterol, high-density lipoprotein cholesterol, low-density lipoprotein cholesterol, and glucose were measured using a Hitachi clinical chemistry autoanalyzer.

### Flow cytometry

Peripheral blood was collected into heparinized tubes. Red blood cells were lysed, and leukocytes were resuspended in staining buffer. Aortas were perfused with PBS via the left ventricle, isolated, cleaned of perivascular adipose tissue, minced, and digested in a tissue dissociation solution (130-110-201, Miltenyi Biotec) at 37 °C with agitation (1,200 rpm) on a magnetic stirrer for 30 min. The resulting cell suspensions were filtered through a 70-μm strainer, centrifuged, and resuspended. To block nonspecific binding to Fc receptor, single-cell suspensions were first incubated with purified anti-mouse CD16/32 (101320, BioLegend, 1:100) at 4 °C for 10 min. Cells were then stained with a live/dead viability dye (Fixable Viability Stain 780, 565388, BD Horizon, 1:750) at 4 °C for 30 min. For neutrophil identification, cells were stained with allophycocyanin-conjugated anti-CD45 (103111, BioLegend, 1:100) and Brilliant Violet 650-conjugated anti-Ly6G (127641, BioLegend, 1:100). To validate neutrophil depletion efficiency while overcoming potential epitope masking by the anti-Ly6G antibody used for neutrophil depletion, an alternative staining panel was employed as described previously, using peridinin chlorophyll protein–cyanine 5.5-conjugated anti-CD11b (101228, BioLegend, 1:100), allophycocyanin-conjugated anti-CD115 (135510, BioLegend, 1:100), and phycoerythrin-conjugated anti-CXCR2 (149304, BioLegend, 1:100) [68]. Samples were acquired on a BD FACS Aria III flow cytometer (BD Biosciences), and data were analyzed using FlowJo software (Tree Star Inc., Ashland, OR, USA).

### FITC-dextran permeability assay

To assess intraplaque microvascular permeability, *Ldlr*^−/−^ mice were intravenously injected with FITC-dextran (Yeasen, 61224ES08) at a dose of 50 mg/kg. One hour later, mice were euthanized and perfusion-fixed with 4% paraformaldehyde. The right carotid artery segment proximal to the proximal stenosis was excised, embedded in optimal cutting temperature compound, and cryosectioned. FITC-dextran extravasation within plaque sections was visualized and imaged using confocal microscopy.

### Immunofluorescence staining

Cryosections were fixed in 4% PFA for 15 min and permeabilized with 0.2% Triton X-100 in PBS for 5 min. After 3 washes with PBS, sections were blocked with 5% goat serum. Sections were then incubated overnight at 4 °C with primary antibodies diluted in blocking buffer. Antibodies used were: anti-MMP9 (ab38898, Abcam, 1:100), anti-α-smooth muscle actin (α-SMA, ab7817, Abcam, 1:200), anti-CD68 (NB100-683, Novus, 1:100), anti-Ly6G (127601, BioLegend, 1:100), anti-4-HNE (PC6313S, Abmart, 1:100), anti-HMOX1 (ab85309, Abcam, 1:100), anti-FTH1 (ab650890, Abcam, 1:100), anti-ETS1 (ET1705-23, HUABIO, 1:100), anti-ACKR1 (ab137044, Abcam, 1:100), anti-VE-cadherin (HA721943, HUABIO, 1:100), anti-TER119 (116202, BioLegend, 1:100), and anti-histone H3 citrullination (ab5103, Abcam, 1:100). Normal isotype immunoglobulin G (IgG) (sc2027, Santa Cruz Biotechnology, 1:100) was used as a negative control. After 3 PBS washes, sections were incubated for 1 h at 37 °C in the dark with the appropriate secondary antibodies: Alexa Fluor 488-conjugated goat anti-rabbit IgG (A-11008, Invitrogen, 1:200), Alexa Fluor 594-conjugated goat anti-rat IgG (A-11007, Invitrogen, 1:200), or Alexa Fluor 594-conjugated goat anti-mouse IgG (A-11005, Invitrogen, 1:200). Nuclei were counterstained with 4′,6-diamidino-2-phenylindole (Sigma-Aldrich, D9542). Images were captured using an LSM710 laser scanning confocal microscope (Carl Zeiss, Germany) and analyzed with ZEN 2009 Light Edition software (Zeiss). Positive staining areas were quantified using ImageJ software (V2.10, NIH, USA).

### Cell culture and stimulations

HUVECs were purchased from Sciencell Research Laboratories (Cat# 8000) and used at passages 2 to 8. HUVECs were cultured in Endothelial Cell Medium (ECM, Cat# 1001, Sciencell) supplemented with 5% fetal bovine serum (FBS) (Cat# 0025, Sciencell), 1% endothelial cell growth supplement (Cat# 1052, Sciencell), and 1% penicillin–streptomycin (Cat# 0503, Sciencell) at 37 °C with 5% CO₂. For stimulation, HUVECs were serum-starved for 6 h and then treated for 12 h with NETs (1 μg/ml), NETs (1 μg/ml) plus DNase I (100 U/ml, Sigma, D7291), or NETs (1 μg/ml) plus the ETS1 inhibitor TK216 (1 μM, MCE, HY-122903). RAW264.7 cells were purchased from Shanghai Zhongqiaoxinzhou Biotechnology (China) and cultured in RPMI 1640 medium with 10% FBS and 1% penicillin–streptomycin. RAW264.7 cells were stimulated with hemin (15 μM, YEASEN, 16009-13-5) or hemin (15 μM) plus Fer-1 (5 μM) for 12 h.

### siRNA knockdown experiments

HUVECs were transfected with 50 nM siRNA targeting human ETS1 or 50 nM control siRNA using Lipofectamine 2000 reagent (11668027, Invitrogen) according to the manufacturer’s instructions. Cells were harvested for downstream experiments 48 h posttransfection.

### ChIP-qPCR

ChIP assays were performed to demonstrate the binding of the ETS1 transcription factor to the promoter regions of VEGFA and VEGFR2 using a Simple ChIP Enzymatic Chromatin IP Kit (Cat# 9003, Cell Signaling Technologies). HUVECs were crosslinked with 1% paraformaldehyde, and the chromatin was fragmented with nuclease and sonication. Then the fragmented chromatin was incubated with anti-ETS1 antibody (ab124282, Abcam, 1:50) or negative control IgG overnight. The next day, chromatin was incubated with protein G magnetic beads. After being washed and purified, precipitated DNA was analyzed by qPCR. Primers used for ChIP-qPCR are listed in Table S5.

### Purification of NETs

HL-60 cells were obtained from Shanghai Zhongqiaoxinzhou Biotechnology (China) and maintained in Iscove’s modified Dulbecco’s medium supplemented with 20% FBS and 1% penicillin–streptomycin. Neutrophil-like differentiation of HL-60 cells was induced by treating cells with 1.25% dimethyl sulfoxide for 5 d. NETs were purified as previously described [69]. Briefly, differentiation of HL-60 cells were stimulated with phorbol 12-myristate 13-acetate (P8139, 100 nM, Sigma-Aldrich) for 3 h. The supernatant (containing NETs) and scraped adherent cells were collected and centrifuged at 450 g for 10 min at 4 °C to remove intact cells. The cell-free supernatant was then centrifuged at 18,000 g for 10 min at 4 °C to pellet NETs. The pellet was resuspended in cold PBS, and the double-stranded DNA concentration was measured to quantify NETs.

### Protein extraction and immunoblotting

Whole-cell lysates were prepared using 1× cell lysis buffer (9803, Cell Signaling Technologies) supplemented with protease inhibitors (04693159001, Roche Molecular Biochemicals). Protein concentration was determined using a BCA protein assay kit. Equal amounts of protein were separated by sodium dodecyl sulfate–polyacrylamide gel electrophoresis and transferred onto polyvinylidene fluoride membranes. Membranes were blocked and then incubated overnight at 4 °C with primary antibodies diluted in blocking buffer. Antibodies used were: anti-SLC7A11 (HA600098, HUABIO, 1:1,000), anti-HMOX1 (ab85309, Abcam, 1:1,000), anti-FTH1 (ab650890, Abcam, 1:1,000), anti-GPX4 (ET1706-45, HUABIO, 1:1,000), anti-VE-cadherin (HA721943, HUABIO, 1:1,000), anti-VEGFA (A0280, Abclonal, 1:1,000), anti-VEGFR2 (9698s, CST, 1:1,000), anti-ETS1 (ET1705-23, HUABIO, 1:1,000), and anti-glyceraldehyde-3-phosphate dehydrogenase (60004-1-Ig, Proteintech, 1:5,000). Protein bands were visualized using chemiluminescence (TANON, China) and imaged with an Amersham Imager 600 system (GE Healthcare, USA).

### RNA-seq

RNA-seq was performed by OE Biotech Co., Ltd. (Shanghai, China). Total RNA was extracted from HUVECs treated with NETs (1.0 μg/ml) or vehicle control using TRIzol reagent (Invitrogen, 15596018). Libraries were constructed from 1.0 μg of total RNA per sample using the Illumina TruSeq RNA Sample Prep Kit (FC-122-1001) and sequenced on an Illumina NovaSeq 6000 platform. Additionally, RNA-seq data for bone marrow-derived macrophages treated with hemin or control were obtained from the Gene Expression Omnibus (GEO) database (accession GSE221734) [24]. For both datasets, DEGs were identified using DESeq2 (v1.22.1). GSEA was performed using the “GSEA” package, and KEGG pathway analysis was conducted using the “clusterProfiler” package. Bulk RNA-seq data and corresponding clinical metadata were downloaded from the GSE163154 dataset [70]. Expression levels of ACKR1, KDR, FABP4, BMX, MGP, and SULF1 were compared between human carotid plaques with and without intraplaque hemorrhage. Single-sample GSEA, implemented in the GSVA package, was used to calculate relative enrichment scores for NETs-related gene signatures [71]. The newly generated RNA-seq data have been deposited in GEO under accession number GSE314216.

### scRNA-seq analyses

scRNA-seq data of human carotid plaques were obtained from the GEO dataset (GSE260657) [25]. Data processing and analysis were performed using the Seurat package in R. Quality control filtering retained cells with mitochondrial gene content < 10%, gene counts between 200 and 4,000, and genes detected in at least 3 cells. Batch correction was applied using the “Harmony” package. Cell clustering was performed with the FindClusters function and visualized via uniform manifold approximation and projection. Cell types were annotated based on canonical marker genes. The AddModuleScore function was used to evaluate the activity of the “tight junction” pathway (REACTOME_TIGHT_JUNCTION_INTERACTIONS.v2025.1.Hs.gmt). DEGs were identified using the FindMarkers function. For Gene Ontology enrichment analysis, DEGs (adjusted *P* < 0.01, |log₂FC| > 1.0) were analyzed using the “clusterProfiler” package.

### Statistical analyses

Statistical analyses for the clinical study were performed using R software (version 4.2.2; The R Foundation for Statistical Computing). Categorical data were presented as frequencies (percentages) and were compared using the chi-square test or Fisher’s exact test, as appropriate. Normally distributed variables were displayed as mean ± SD and were analyzed with the Student *t* test. Non-normally distributed variables were reported as median (interquartile range) and were compared using the Mann–Whitney U test. Multivariate logistic regression models were used to compute adjusted ORs with 95% CIs, adjusting for age, sex, body mass index, smoking, hypertension, history of stroke, and diabetes. Mediation analysis was conducted using the “Mediation” package to explore the role of white blood cell count, neutrophil count, and lymphocyte count in the relationship between MI and plaque progression. In this model, MI was the independent variable, plaque progression was the dependent variable, and each immune cell type (white blood cell count, neutrophil count, and lymphocyte count) was the mediator. The effect size for each mediator was expressed as the ratio of the indirect effect to the total effect. A 2-sided *P* value < 0.05 was considered statistically significant.

For MR analyses, causal estimates were derived using the “TwoSampleMR” package in R. The primary analysis employed the IVW method. Sensitivity analyses were performed using the MR Egger, weighted median, simple mode, and weighted mode methods. The IVW method combines Wald ratios for all included SNPs under the assumption that all are valid instruments. Results were reported as ORs with 95% CIs.

Experimental data from in vitro and in vivo studies were presented as means ± SD from at least 3 biologically independent replicates. Comparisons between 2 groups were performed using the 2-tailed Student *t* test (for normally distributed data) or the Mann–Whitney U test (for non-normally distributed data). Comparisons among 3 or more groups were conducted using 1-way or 2-way analysis of variance (ANOVA; for normally distributed data) followed by appropriate post hoc tests, or the Kruskal–Wallis test (for non-normally distributed data). Pearson correlation analysis was used to assess associations between variables. A 2-sided *P* value < 0.05 was considered statistically significant.

## Data Availability

Data supporting the results of this study can be obtained from corresponding authors upon reasonable request.
