# Supplementary material for: Neutrophil Extracellular Traps-Mediated Intraplaque Angiogenesis and Microvascular Permeability Promote Rapid Plaque Progression following Myocardial Infarction
Source: Research (Wash D C). 2026 Oct 19;9:1451. doi: 10.34133/research.1451 (PMC13678088; doi:10.34133/research.1451)
Supplement: Supplementary file 1 [file research.1451.f1.docx]

**SUPPLEMENTARY MATERIALS**

**Supplementary materials and methods**

EdU Assay

Cell proliferation was assessed using the Yefluor 594 EdU Imaging Kit (Cat# 40276ES60, Yeasen) according to the manufacturer’s instructions. Nuclei were counterstained with DAPI. Images of EdU-positive cells were captured using a Leica DMI6000 microscope. Three to four random fields were analyzed for each experimental group. The proliferation rate was calculated as the percentage of EdU-positive cells relative to the total number of DAPI-stained nuclei.

Cell Counting Kit-8 (CCK-8) assay

Cell viability was assessed by the CCK-8 assay. Briefly, cells were seeded into a 96-well plate at a density of 2×10^3^ cells per well, with four replicates for each group. The cells were treated with different concentrations of NETs. After 24 hours, a 10 μl CCK-8 reagent (HY-K0301, MCE, USA) was added according to the manufacturer’s instructions, followed by a 2-hour incubation. The absorbance was measured at 450 nm using a microplate reader.

Migration assay

A scratch-wound assay was performed to evaluate cell migration. HUVECs were seeded in 6-well plates and grown to 100% confluence. A linear wound was created using a sterile pipette tip. Images (n = 4 per well) were captured immediately (0 h) and at 24 h after wounding using a Leica DMI6000 microscope. The migrated area was calculated as the difference between the initial wound area (S₀) and the remaining wound area (Sₜ). The migration rate was expressed as a percentage: Migration rate (%) = [(S₀ − Sₜ) / S₀] × 100.

Tube formation assay

Matrigel (356234, BD-Pharmingen, USA) was diluted 1:1 with DMEM and added to 24-well plates. Plates were incubated at 37°C for 30 minutes to allow gel polymerization. HUVECs (1 × 10⁴ cells/well) were resuspended in the indicated conditioned media, seeded onto the Matrigel-coated wells, and incubated in growth factor–free medium at 37°C for 6 hours. Tube formation was observed under an inverted microscope, and the total tube length was quantified using ImageJ software (V2.10, NIH, USA).

Evans Blue permeability assay

HUVECs were seeded in Transwell chambers inserted into a 24-well cell culture plate. Complete medium was added to the lower chamber in advance, and the plate was incubated at 37 °C in a 5% CO₂ cell incubator until a confluent cell monolayer was formed. The Transwell chambers were then transferred to a new 24-well plate. A 4% BSA solution was added to the lower chamber, and a mixture of 2% Evans Blue and 4% BSA (final concentration 0.67 mg/mL) was added to the upper chamber to ensure equal liquid levels. The plate was incubated at 37 °C in a 5% CO₂ cell incubator for 1 hour. The chambers were removed, and the liquid from the lower chamber was transferred to a 96-well plate. The OD value of each group was measured at a wavelength of 620 nm using a microplate reader.

Quantification of lipid peroxidation

The culture medium was discarded, and the cells were dissociated using trypsin. The cell suspension was then centrifuged at 1000g for 5 minutes at 4°C. After discarding the supernatant, the cells were washed twice with PBS. According to the manufacturer’s instructions, 5 µM of BODIPY 581/591 C11 (D3861, Thermo Fisher Scientific) was added, and the cells were incubated at room temperature for 30 minutes in the dark. Next, the cells were centrifuged at 400g for 3 minutes at 4°C, washed twice again, and resuspended in PBS for analysis by flow cytometry.

Quantification of cell death

The culture medium was discarded, and the cells were dissociated with trypsin. After centrifugation at 1000g for 5 minutes, the cell pellet was collected and washed. According to the manufacturer’s instructions (HY‑K1073, MedChemExpress), Binding Buffer, PI Stain were sequentially added. The cells were then incubated at room temperature in the dark for 15 minutes, followed by detection of fluorescence intensity using flow cytometry.

**Supplementary figures**

***Fig. S1.*** ***MI promotes rapid plaque progression in non-culprit arteries without altering body weight, lipid profile or glucose levels.*** *A, B) Assessment of body weight, lipid profile and glucose levels in the sham and MI groups (n = 8 per group). C) Representative images of Masson’s trichrome staining, and quantification of luminal stenosis (%) and collagen content in the atherosclerotic lesions from aortic arch, thoracic aorta, and abdominal aorta at 4 weeks post-surgery (Scale bar = 100 µm, n = 6 per group). D) Representative images of intraplaque MMP9, α-SMA and CD68 immunofluorescence staining in BCA lesions from the sham and MI groups at 4 weeks post-surgery (Scale bar = 100 µm). E, F, G) Quantification of MMP9^+^, α-SMA^+^ and CD68^+^ area relative to plaque area in D (n = 6 per group). Statistical analyses were performed using two-way ANOVA test (A) and unpaired Student’s t-test (B, C, E, F and G).*

***Fig. S2.*** ***Increased neutrophil infiltration and NETs formation in non-culprit lesions after MI.*** *A) Flow cytometry gating strategy applied in Fig. 2F. B) Representative immunofluorescence images of Ly6G and H3CIT co-staining, and quantification of Ly6G^+^ area and Ly6G^+^H3CIT^+^ area relative to plaque area in the atherosclerotic lesions from aortic arch, thoracic aorta, and abdominal aorta at 4 weeks post-surgery (Scale bar = 100 µm, n = 6 per group). C) Correlation analyses of Ly6G^+^ area and Ly6G^+^H3CIT^+^ area with MMP9^+^, α-SMA^+^ and CD68^+^ area in BCA lesions. Statistical analyses were performed using unpaired Student’s t-test (B) and Pearson correlation analysis (C).*

***Fig. S3. Genetic inhibition of NETs formation attenuates MI-induced rapid plaque progression in non-culprit lesions.*** *A) Flow cytometry analysis to verify the efficiency of neutrophil depletion in peripheral blood using the canonical (CD45⁺CD11b⁺CD115⁻Ly6G⁺) and alternative (CD45⁺CD11b⁺CD115⁻CXCR2⁺) gating strategies (n = 4 per group). B) Schematic of the neutrophil adoptive transfer experiment. C) Representative images of Masson’s trichrome staining, MMP9, α-SMA and CD68 immunofluorescence staining across the indicated experimental groups (Scale bar = 100 µm). D, E) Quantification of luminal stenosis (%) and collagen content from the Masson’s trichrome staining in C (n = 6 per group). F, G, H) Quantification of MMP9^+^, α-SMA^+^ and CD68^+^ area relative to plaque area in C (n = 6 per group). Statistical analyses were performed using Mann-Whitney U (A) and one-way ANOVA with Bonferroni post-test (D-H).*

***Fig. S4. NETs-mediated intraplaque hemorrhage drives rapid progression of non-culprit plaques following MI, potentially via macrophage ferroptosis.*** *A) Correlation analysis of Ly6G^+^H3CIT^+^ area with TER119^+^ area and Prussian blue-positive area in BCA lesions. B) Representative images and quantification of TER119 immunofluorescence staining in BCA lesions from the control and DNase I groups at 4-weeks post-MI (Scale bar = 100 µm, n = 6 per group). C) Representative images and quantification of Prussian blue staining in BCA lesions from the control and DNase I groups at 4 weeks post-MI (Scale bar = 50 µm, n = 6 per group). D) DEGs in hemin-treated BMDMs from the published RNA-seq dataset (GSE221734). E, F) Representative images and quantification of CD68^+^HMOX1^+^ and CD68^+^FTH1^+^ immunofluorescence staining in BCA lesions from the sham and MI groups at 4 weeks post-surgery (Scale bar = 100 µm, n = 6 per group). G, H) Representative images and quantification of CD68^+^HMOX1^+^ and CD68^+^FTH1^+^ immunofluorescence staining in BCA lesions from the control and DNase I groups at 4 weeks post-MI (Scale bar = 100 µm, n = 6 per group). Statistical analyses were performed using Pearson correlation analysis (A) and unpaired Student’s t-test (B, C, E, F, G and H).*

***Fig. S5. Ferroptosis inhibition attenuates rapid plaque progression in non-culprit lesions following MI.*** *A) Schematic of the experimental timeline for ferroptosis inhibition. B) Representative Masson’s trichrome staining images and corresponding quantification of luminal stenosis (%) and collagen content in BCA lesions from the control and Fer-1 groups (Scale bar = 100 µm, n = 6 per group). C) Representative images of intraplaque MMP9, α-SMA and CD68 immunofluorescence staining in BCA lesions from the control and Fer-1 groups (Scale bar = 100 µm). D) Quantification of MMP9^+^, α-SMA^+^ and CD68^+^ area relative to plaque area in BCA lesions from the control and Fer-1 groups in C (n = 6 per group). Statistical analyses were performed using unpaired Student’s t-test (B and D).*

***Fig. S6.*** ***Analyses of scRNA-seq dataset of human carotid plaques.*** *A, B) Uniform manifold approximation and projection (UMAP) visualization and dotplot of marker gene expression for scRNA-seq data from human carotid plaques (GSE260657). C, D) Dotplot and featureplot of markers defining the two distinct EC subpopulations. E) GO enrichment analysis of upregulated DEGs in EC1 and EC2. F)  Comparison of tight junction pathway scores between EC1 and EC2. G) Expression levels of ACKR1, KDR, FABP4, BMX, MGP, and SULF1 in plaques with or without intraplaque hemorrhage in bulk RNA‑seq data (GSE163154). Statistical analyses were performed using Mann–Whitney U test (F and G).*

***Fig. S7. MI promotes rapid plaque progression in TS model through NETs formation.*** *A) Representative images of Masson’s trichrome staining and immunofluorescence staining for intraplaque MMP9, α‑SMA, CD68, TER119, and 4‑HNE across the indicated experimental groups (Scale bar = 100 µm for all panels). B, C) Quantification of luminal stenosis (%) and collagen content from the Masson’s trichrome staining in A (n = 5-6 per group). D, E, F, G, H) Quantification of MMP9^+^, α-SMA^+^, CD68^+^, TER119^+^ and 4-HNE^+^ area relative to plaque area in A (n = 5-6 per group). Statistical analyses were performed using one-way ANOVA with Bonferroni post-test (B-H).*

***Fig. S8.*** ***NETs promote angiogenesis and endothelial permeability in vitro.*** *A) CCK‑8 assay of HUVECs treated with graded concentrations of NETs (n=4 per group). B) Representative images and quantification of EDU staining of HUVECs treated with graded concentrations of NETs (Scale bar = 100 µm, n = 3 per group). C) Representative images and quantification of migration assay in HUVECs treated with graded concentrations of NETs (Scale bar = 100 µm, n = 3 per group). D) Representative images and quantification of tube formation assay in HUVECs treated with graded concentrations of NETs (Scale bar = 50 µm, n = 4 per group). E) Evans-blue cell permeability analysis of HUVECs treated with graded concentrations of NETs (n=5 per group). F) Immunoblotting and quantification of the relative VE-cadherin protein levels in HUVECs treated with graded concentrations of NETs (n = 3 per group). G) Representative images and quantification of EDU staining across the indicated groups (Scale bar = 100 µm, n = 3 per group). H) Representative images and quantification of the migration assay across the indicated groups (Scale bar = 100 µm, n = 3 per group). Statistical analyses were performed using one-way ANOVA with Bonferroni post-test (A-H).*

***Fig. S9.*** ***NETs promote angiogenesis and endothelial permeability via ETS1-mediated VEGFA/VEGFR2 signaling pathway.*** *A, B) DEGs identified by RNA-seq in HUVECs treated with NETs. C) Representative images and quantification of intraplaque ETS1 immunofluorescence staining across the indicated groups (Scale bar = 100 µm, n = 5-6 per group). D) Representative images and quantification of EDU staining across the indicated groups (Scale bar = 100 µm, n = 3 per group). E) Representative images and quantification of the migration assay across the indicated groups (Scale bar = 100 µm, n = 3 per group). Statistical analyses were performed using one-way ANOVA with Bonferroni post-test (C-E).*

***Fig. S10.*** ***TK216 attenuates NETs-induced angiogenesis and endothelial permeability in vitro.*** *A) Immunoblotting and quantification of the relative protein levels of VEGFA, VEGFR2, and ETS1 across the indicated groups (n = 4 per group). B) The occupancy of ETS1 at the promoter regions of VEGFA and VEGFR2 was determined by ChIP-qPCR across the indicated groups (n=3 per group). C) Representative images and quantification of EDU staining across the indicated groups (Scale bar = 100 µm, n = 3 per group). D) Representative images and quantification of the migration assay across the indicated groups (Scale bar = 100 µm, n = 3 per group). E) Representative images and quantification of the tube formation assay across the indicated groups (Scale bar = 50 µm, n = 4 per group). F) Evans-blue cell permeability analysis across the indicated groups (n = 5 per group). G) Immunoblotting and quantification of the relative VE-cadherin protein levels across the indicated groups (n = 4 per group). Statistical analyses were performed using one-way ANOVA with Bonferroni post-test (A, C-G) and two-way ANOVA with Bonferroni post-test (B).*

***Fig. S11.*** ***TK216 attenuates rapid plaque progression following MI in the TS model.*** *A) Representative images of Masson’s trichrome staining and immunofluorescence staining for intraplaque MMP9, α‑SMA, CD68, TER119, and 4‑HNE across the indicated experimental groups (Scale bar = 100 µm for all panels). B, C) Quantification of luminal stenosis and collagen content from the Masson’s trichrome staining in A (n = 5 per group). D, E, F, G, H) Quantification of MMP9^+^, α-SMA^+^, CD68^+^, TER119^+^ and 4-HNE^+^ area relative to plaque area in A (n = 5 per group). Statistical analyses were performed using one-way ANOVA with Bonferroni post-test (B-G).*

***Fig. S12. Single-sample gene set enrichment analysis (ssGSEA) in the RNA seq dataset (GSE163154).*** *A) NETs-related score in plaques with and without intraplaque hemorrhage based on bulk RNA-seq data (GSE163154). B) Correlation analyses of NETs-related score with EC1 markers (ACKR1, KDR, FABP4), EC2 markers (BMX, MGP, SULF1), and plaque instability markers (MMP9, CD68, ACTA2). Statistical analyses were performed using the Mann-Whitney U test (A) and Pearson correlation analysis (B).*

**Supplementary tables**

***Table S1.*** *Characteristics of the 42 candidate SNPs for MI.*

| SNP | effect_allele | other_allele | beta | se | eaf | pval | F |
| --- | --- | --- | --- | --- | --- | --- | --- |
| rs10056700 | T | C | -0.0622277 | 0.010931 | 0.331553 | 1.25E-08 | 32.40748711 |
| rs11591147 | G | T | -0.268899 | 0.0287704 | 0.0364805 | 9.07E-21 | 87.35428659 |
| rs11619038 | C | T | 0.0887032 | 0.0156639 | 0.118764 | 1.49E-08 | 32.06833547 |
| rs117733303 | A | G | 0.36443 | 0.0473007 | 0.0106573 | 1.31E-14 | 59.35957096 |
| rs118039278 | G | A | 0.276023 | 0.0232471 | 0.0456111 | 1.63E-32 | 140.9777656 |
| rs11970276 | A | G | 0.0659112 | 0.0120799 | 0.230537 | 4.86E-08 | 29.77072465 |
| rs12052058 | G | T | -0.105933 | 0.0126592 | 0.211511 | 5.86E-17 | 70.02412128 |
| rs12324886 | A | G | -0.0798219 | 0.0106298 | 0.636876 | 5.95E-14 | 56.38863509 |
| rs12713468 | G | A | -0.0627048 | 0.010707 | 0.356858 | 4.73E-09 | 34.29759088 |
| rs137943060 | G | A | 0.228855 | 0.0374205 | 0.018402 | 9.61E-10 | 37.40238406 |
| rs142816409 | A | G | 0.0920505 | 0.0168261 | 0.0995047 | 4.48E-08 | 29.9283669 |
| rs1476382 | C | T | 0.0908355 | 0.0131027 | 0.182449 | 4.13E-12 | 48.06038817 |
| rs16986953 | G | A | 0.107007 | 0.0192543 | 0.0730183 | 2.74E-08 | 30.88634766 |
| rs17026396 | T | C | 0.0657861 | 0.0103353 | 0.425757 | 1.95E-10 | 40.51536484 |
| rs17489268 | T | A | -0.0716171 | 0.0119615 | 0.246149 | 2.13E-09 | 35.84757813 |
| rs1909195 | T | C | 0.0968957 | 0.0128872 | 0.799027 | 5.53E-14 | 56.53137112 |
| rs2107595 | G | A | 0.0869981 | 0.0128895 | 0.192769 | 1.48E-11 | 45.55594303 |
| rs2246828 | G | A | 0.0644247 | 0.0106785 | 0.350582 | 1.61E-09 | 36.39837146 |
| rs2811711 | T | C | 0.0928134 | 0.0166064 | 0.103497 | 2.28E-08 | 31.23690684 |
| rs28451064 | G | A | 0.1324 | 0.0139502 | 0.153157 | 2.29E-21 | 90.07675848 |
| rs28673728 | A | G | 0.057635 | 0.0102403 | 0.538762 | 1.82E-08 | 31.67706109 |
| rs35574960 | C | T | -0.0984868 | 0.0160812 | 0.11712 | 9.11E-10 | 37.50738566 |
| rs3918226 | C | T | 0.142092 | 0.0196692 | 0.0697918 | 5.04E-13 | 52.1871414 |
| rs3927760 | C | A | 0.101432 | 0.0134893 | 0.16734 | 5.50E-14 | 56.54169164 |
| rs415434 | A | G | -0.0559179 | 0.0102555 | 0.523 | 4.97E-08 | 29.72936789 |
| rs4632638 | G | A | -0.083824 | 0.0124063 | 0.786871 | 1.41E-11 | 45.65095337 |
| rs4948807 | T | A | 0.0773895 | 0.0111538 | 0.693907 | 3.96E-12 | 48.14110148 |
| rs55845370 | A | G | 0.061997 | 0.0105013 | 0.383634 | 3.55E-09 | 34.85401914 |
| rs55965782 | T | C | 0.141785 | 0.0151354 | 0.126123 | 7.41E-21 | 87.75470609 |
| rs565898017 | G | T | -0.323508 | 0.0321667 | 0.028932 | 8.53E-24 | 101.1473896 |
| rs639750 | G | T | 0.0679972 | 0.0103991 | 0.583662 | 6.20E-11 | 42.75512665 |
| rs642803 | C | T | -0.0572536 | 0.0104891 | 0.396963 | 4.80E-08 | 29.79386148 |
| rs646776 | C | T | 0.0781937 | 0.0125119 | 0.784674 | 4.12E-10 | 39.05661899 |
| rs653178 | C | T | -0.068887 | 0.0103527 | 0.585262 | 2.85E-11 | 44.27564862 |
| rs7213347 | G | C | 0.0755209 | 0.0112965 | 0.707703 | 2.30E-11 | 44.6934775 |
| rs72934535 | T | C | 0.122969 | 0.0171694 | 0.0954187 | 7.95E-13 | 51.29543208 |
| rs7412 | C | T | -0.232693 | 0.023376 | 0.0536119 | 2.41E-23 | 99.08864449 |
| rs7668383 | T | C | -0.0843811 | 0.0133895 | 0.180641 | 2.94E-10 | 39.71543219 |
| rs9349379 | A | G | 0.136873 | 0.0102007 | 0.451153 | 4.74E-41 | 180.041768 |
| rs9515203 | T | C | -0.0933061 | 0.0116894 | 0.26215 | 1.44E-15 | 63.71376676 |
| rs9932382 | T | C | 0.0657869 | 0.0112595 | 0.700263 | 5.13E-09 | 34.13801624 |
| rs9989421 | A | G | 0.0745065 | 0.0119268 | 0.750897 | 4.18E-10 | 39.02456752 |

***Table S2:*** *MR analyses for the association of MI with atherosclerosis.*

|  | method | nsnp | P value | OR (95% CI） |
| --- | --- | --- | --- | --- |
| Atherosclerosis | MR Egger | 39 | 0.213793086 | 1.484 (0.805-2.733) |
|  | Weighted median | 39 | 0.046501532 | 1.312 (1.004-1.714) |
|  | Inverse variance weighted | 39 | 0.000936081 | 1.469 (1.170-1.846) |
|  | Simple mode | 39 | 0.250041187 | 1.346 (0.818-2.214) |
|  | Weighted mode | 39 | 0.295543957 | 1.237 (0.835-1.831) |
|  |  |  |  |  |
| Coronary atherosclerosis | MR Egger | 22 | 0.512836824 | 1.187 (0.717-1.967) |
|  | Weighted median | 22 | 1.81E-15 | 1.718 (1.503-1.963) |
|  | Inverse variance weighted | 22 | 3.46E-15 | 1.685 (1.480-1.918) |
|  | Simple mode | 22 | 1.42E-05 | 1.776 (1.453-2.170) |
|  | Weighted mode | 22 | 2.88E-06 | 1.723 (1.455-2.039) |
|  |  |  |  |  |
| Cerebral atherosclerosis | MR Egger | 40 | 0.921163693 | 0.949 (0.336-2.679) |
|  | Weighted median | 40 | 0.733852033 | 0.900 (0.492-1.649) |
|  | Inverse variance weighted | 40 | 0.08540193 | 1.468 (0.948-2.274) |
|  | Simple mode | 40 | 0.066191188 | 3.906 (0.951-16.043) |
|  | Weighted mode | 40 | 0.356993082 | 0.625 (0.232-1.680) |
|  |  |  |  |  |
| Atherosclerosis of aorta | MR Egger | 40 | 0.007478636 | 3.342 (1.447-7.717) |
|  | Weighted median | 40 | 0.01828035 | 1.912 (1.116-3.277) |
|  | Inverse variance weighted | 40 | 0.000179053 | 1.960 (1.378-2.787) |
|  | Simple mode | 40 | 0.197398688 | 1.977 (0.714-5.473) |
|  | Weighted mode | 40 | 0.196044902 | 1.946 (0.722-5.250) |
|  |  |  |  |  |
| Atherosclerosis of the extremities | MR Egger | 40 | 0.017402038 | 2.335 (1.197-4.555) |
|  | Weighted median | 40 | 0.013827334 | 1.570 (1.096-2.248) |
|  | Inverse variance weighted | 40 | 0.000145624 | 1.718 (1.299-2.271) |
|  | Simple mode | 40 | 0.306742196 | 1.495 (0.698-3.201) |
|  | Weighted mode | 40 | 0.252628379 | 1.495 (0.758-2.948) |
|  |  |  |  |  |
| Atherosclerosis of renal artery | MR Egger | 40 | 0.807137407 | 1.220 (0.250-5.940) |
|  | Weighted median | 40 | 0.435505808 | 0.698 (0.282-1.725) |
|  | Inverse variance weighted | 40 | 0.928881212 | 1.031 (0.533-1.994) |
|  | Simple mode | 40 | 0.697999483 | 0.693 (0.110-4.354) |
|  | Weighted mode | 40 | 0.646337685 | 0.693 (0.147-3.276) |

***Table S3:*** *Baseline clinical characteristics and biochemical assessments of populations.*

|  | *Stable CAD n=50* | *MI n=100* | *P value* |
| --- | --- | --- | --- |
| *Male* | *34 (68.0%)* | *80 (80.0%)* | *0.156* |
| *Age, years* | *66.5±8.98* | *64.3±9.73* | *0.176* |
| *Smoking* | *21 (42.0%)* | *51 (51.0%)* | *0.386* |
| *Hypertension* | *40 (80.0%)* | *70 (70.0%)* | *0.267* |
| *Hyperlipidemia* | *10 (20.0%)* | *28 (28.0%)* | *0.388* |
| *Diabetes* | *14 (28.0%)* | *26 (26.0%)* | *0.948* |
| *History of stroke* | *7 (14.0%)* | *19 (19.0%)* | *0.593* |
| *BMI, kg/m2* | *24.6±3.14* | *24.5±3.78* | *0.920* |
| *Fasting plasma glucose, mmol/L* | *6.00±2.48* | *5.87±1.43* | *0.724* |
| *Total cholesterol, mmol/L* | *4.86±0.83* | *4.82±1.31* | *0.795* |
| *Triglyceride, mmol/L* | *1.91±0.81* | *2.04±2.03* | *0.565* |
| *HDL-C, mmol/L* | *1.19±0.26* | *1.11±0.29* | *0.092* |
| *LDL-C, mmol/L* | *2.88±0.71* | *2.79±0.91* | *0.536* |
| *CRP, mg/ml* | *10.9 (7.05-16.1)* | *15.6 (7.75-22.0)* | *0.025* |
| *Neutrophils, ×109/L* | *4.18±1.25* | *5.04±1.83* | *0.001* |

***Table S4:*** *Angiographic assessments of populations.*

|  | Stable CAD n=50 | MI n=100 | P value |
| --- | --- | --- | --- |
| Location of target lesion at baseline |  |  | 0.533 |
| LAD | 18 (36.0%) | 33 (33.0%) |  |
| LCX | 14 (28.0%) | 37 (37.0%) |  |
| RCA | 18 (36.0%) | 30 (30.0%) |  |
| Location of analyzed lesion at follow-up |  |  | 0.344 |
| LAD | 19 (38.0%) | 34 (34.0%) |  |
| LCX | 15 (30.0%) | 35 (35.0%) |  |
| RCA | 16 (32.0%) | 31 (31.0%) |  |
| Baseline CAG |  |  |  |
| Diameter stenosis, % | 35.0 (26.0-44.0) | 35.0 (26.0-44.0) | 0.943 |
| MLD, mm | 1.95 (1.50-2.50) | 1.90 (1.58-2.42) | 0.914 |
| Follow-up CAG |  |  |  |
| Diameter stenosis, % | 34.0 (28.0-45.0) | 41.0 (33.0-54.0) | 0.004 |
| MLD, mm | 2.00 (1.40-2.48) | 1.60 (1.20-2.20) | 0.051 |
| Change of diameter stenosis, % | -1.30 (-7.15-1.90) | -6.45 (-15.42- -0.15) | 0.007 |
| Change of MLD, mm | 0.10 (-0.10 - 0.20) | 0.20 (-0.10-0.40) | 0.015 |
| Plaque progression | 7 (14.0%) | 37 (37.0%) | 0.006 |

***Table S5: Primers used for ChIP-qPCR.***

| Gene |  | Primer |
| --- | --- | --- |
| VEGFR2 | Forward | CAGCTCCCCAAGCCCATTTA |
| VEGFR2 | Reverse | CGCCCGTTACCGAGTACTTT |
| VEGFA | Forward | GAGGAGGAAGAAGAGAAGGA |
| VEGFA | Reverse | AAAGTTCATGGTTTCGGAGG |
